# Mechanism Across Scales: A Holistic Modeling Framework Integrating Laboratory and Field Studies for Microbial Ecology

**DOI:** 10.3389/fmicb.2021.642422

**Published:** 2021-03-24

**Authors:** Lauren M. Lui, Erica L.-W. Majumder, Heidi J. Smith, Hans K. Carlson, Frederick von Netzer, Matthew W. Fields, David A. Stahl, Jizhong Zhou, Terry C. Hazen, Nitin S. Baliga, Paul D. Adams, Adam P. Arkin

**Affiliations:** ^1^Division of Environmental Genomics and Systems Biology, Lawrence Berkeley National Laboratory, Berkeley, CA, United States; ^2^Department of Bacteriology, University of Wisconsin–Madison, Madison, WI, United States; ^3^Center for Biofilm Engineering, Department of Microbiology and Immunology, Montana State University, Bozeman, MT, United States; ^4^Department of Civil and Environmental Engineering, University of Washington, Seattle, WA, United States; ^5^Institute for Environmental Genomics, Department of Microbiology & Plant Biology, School of Civil Engineering and Environmental Sciences, University of Oklahoma, Norman, OK, United States; ^6^Department of Civil and Environmental Engineering, University of Tennessee, Knoxville, Knoxville, TN, United States; ^7^Institute for Systems Biology, Seattle, WA, United States; ^8^Department of Bioengineering, University of California, Berkeley, Berkeley, CA, United States

**Keywords:** reactive transport modeling, metabolic model, species interaction network, systems biology, subsurface microbial ecology

## Abstract

Over the last century, leaps in technology for imaging, sampling, detection, high-throughput sequencing, and -omics analyses have revolutionized microbial ecology to enable rapid acquisition of extensive datasets for microbial communities across the ever-increasing temporal and spatial scales. The present challenge is capitalizing on our enhanced abilities of observation and integrating diverse data types from different scales, resolutions, and disciplines to reach a causal and mechanistic understanding of how microbial communities transform and respond to perturbations in the environment. This type of causal and mechanistic understanding will make predictions of microbial community behavior more robust and actionable in addressing microbially mediated global problems. To discern drivers of microbial community assembly and function, we recognize the need for a conceptual, quantitative framework that connects measurements of genomic potential, the environment, and ecological and physical forces to rates of microbial growth at specific locations. We describe the Framework for Integrated, Conceptual, and Systematic Microbial Ecology (FICSME), an experimental design framework for conducting process-focused microbial ecology studies that incorporates biological, chemical, and physical drivers of a microbial system into a conceptual model. Through iterative cycles that advance our understanding of the coupling across scales and processes, we can reliably predict how perturbations to microbial systems impact ecosystem-scale processes or vice versa. We describe an approach and potential applications for using the FICSME to elucidate the mechanisms of globally important ecological and physical processes, toward attaining the goal of predicting the structure and function of microbial communities in chemically complex natural environments.

## Introduction

Microbial communities serve critical roles in all ecosystems and have a profound impact on human health, environmental health, and industrial capabilities. As such, it is desirable to have robust, actionable directions for intervention of microbial community function. However, the multiscale, stochastic, spatio-temporal, and diverse nature of microbial processes makes it difficult to achieve predictive understanding of microbial systems, despite the large body of microbial ecology research. This disconnect between basic and translational science in microbial ecology stems largely from the intractability of most microbes and microbial communities—*in situ* in their natural habitat and in the laboratory, due to challenges with cultivation and genetic manipulation. As a result, most of our understanding of microbial ecology is patchwork, synthesized from model microbes that often do not represent the full set of capabilities of the microbial communities associated with real-world phenomena. Many hurdles preventing the direct investigation of microbial communities have recently been overcome with the integration of technologies that combine *in situ* monitoring, high-throughput culturing, genetic manipulation, multi-omics profiling, predictive computational modeling, and microbiome engineering to test hypotheses in a natural context. Microbial ecology is ready to shift to making basic and translational science a continuum, instead of two disconnected silos. To bridge basic science and actionable results, microbial ecologists are calling for a movement toward testable models, integration of experiment and theory, and focused hypothesis-driven studies (Zhou, 2009; Widder et al., 2016; Prosser, 2020; Prosser and Martiny, 2020).

The rigor of modeling frameworks allows us to formally define what observations are necessary to support conclusions and to make and understand the quality of our predictions, thus directing experimental design, efficient data collection, and paths for intervention. Models tie the components of a system (e.g., genes, species, communities, and chemicals) with relationships that can represent the system state (Stokes and Arkin, 2007). Thus, modeling forces investigators to evaluate what measurements need to be taken of the system and what the assumed relationships are between the system components (Lopatkin and Collins, 2020). Models are a simplification of reality that ideally enable scientists to predict how perturbations will influence a system and expose errors in proposed theories (Nordstrom and Kirk Nordstrom, 2012; Wagner, 2015).

Modeling is not new to microbial ecology (Box 1), but models are often confined to one scale or experimental system, such as *in situ* field studies, mesocosms, or focused isolate studies. Microbial ecology models that bridge scales from genes to ecosystems are rare due to the diverse expertise and data collection required (Scheibe et al., 2009; Zhuang et al., 2011; Guo et al., 2018, 2020; Gao et al., 2020; Ning et al., 2020) but are needed to help bridge basic and translational science for microbial ecology. Predictive understanding of how microbial communities respond to their environment requires mechanistic knowledge of relationships and interactions between genes, organisms, environmental chemistry, and physical processes of the system. Modeling efforts in microbial ecology are currently lacking a standardized iterative approach that can accommodate research progress in both field and detailed laboratory investigations. Iterative approaches between experiment and modeling across scales are more standardized in reactive transport modeling (Maher and Mayer, 2019; Meile and Scheibe, 2019) and systems biology of the cell (Arkin and Schaffer, 2011; Lopatkin and Collins, 2020). Such standardized iterative reaction or process modeling is also common in chemistry and structural biology, among other fields. Methodologies include molecular dynamics, Monte Carlo simulations, and density functional theory (van Mourik et al., 2014). Molecular dynamics, in particular, could inform modeling standards in microbial ecology for modeling kinetics, thermodynamics, and Brownian motion of thousands of different molecules simultaneously while accounting for environmental conditions like pH, temperature, and concentration. Molecular dynamics samples reaction landscapes and identifies causal factors that drive reactions to different endpoints (Venable et al., 2019). While molecular dynamics simulations are beyond the scope of microbial ecology, such modeling techniques from other fields can offer insights on how to effectively improve standards. Working in an iterative continuum will ultimately push microbial ecology forward with the ability to conduct robust, predictive studies.

**Box 1. Commonly used models in microbial ecology.**

Models are used to quantitatively describe variables of interest and for the presented framework are used to provide insight and structure to address unanswered microbial ecology questions (Box 2). Below are commonly used deterministic modeling approaches that have been incorporated into the proposed framework.

• *Genome-scale metabolic models* aim to reconstruct an organism’s metabolic networks based on gene content. Metabolic models predict the physiological response of organisms to fluctuations over a range of environmental conditions based on genetic potential and the flow of metabolites through metabolic networks. A prerequisite for these models is to have the genome sequences for the organisms of interest, and limitations of these models include gathering enough data for proper parameterization (including flux constraints and thermodynamics), gene function prediction, and ability to validate the models.
• *Species interaction models* are used to infer networks of interactions between microorganisms and represent processes occurring at the community scale. A common example of such a model is the generalized Lotka–Volterra (gLV) models, which incorporate species interactions into dynamic models and can be used to evaluate species interactions. Limitations of species interaction models are that it is challenging to acquire meaningful and representative species interaction data, especially from natural environments, and that it is difficult to validate relationships inferred via multiple regression. There is a need for additional targeted multivariate approaches that are focused on identifying significant interactions and directionality from complex relationships.
• *Reactive transport models* (RTMs) span larger ecosystem-scale processes and are used to predict the distribution of specific compounds over time and space with the overall goal of providing a conceptual framework to understand the factors that control biotic and abiotic transformations of chemical constituents over space and time. RTMs are partial differential equation models wherein the variables of the model—chemical or species abundances—are functions of time and space, and changes in these are driven by transport and chemical processes. This allows models of dispersal, attachment, and feedback on spatial aspects of environment to be incorporated. Similar to other modeling approaches, the level of detail incorporated into RTM has the potential to drastically influence the outcome of the model. Initially, RTM did not include microbes; however, more recently RTM have evolved to incorporate microbially mediated processes as rate expressions dependent on the concentration of substrates (expressed as first-order kinetics) and electron acceptors (expressed through additional Michaelis–Menten terms) (Meile and Scheibe, 2019; Zelaya et al., 2019, references within). RTMs that use rate expressions to represent microbial processes do not necessarily identify individual microbes responsible for specific chemical transformations.

To aid efforts that are building predictive understanding from genes to ecosystems, we have developed a conceptual modeling framework that models the composition, function, and ecological processes of microbial communities and environmental components at different scales (e.g., genes, individuals, populations, communities, and ecosystems) combined into an encompassing continuum between field and laboratory studies. This framework incorporates the foundational work that others have done to model microbial ecology processes (Box 1) and will help microbial ecologists (1) develop hypotheses, (2) determine measurements needed for focused sampling, laboratory efforts, and reduced analytical burdens, (3) discern processes to capture in their study, (4) incorporate results from other studies, and (5) plan long-term projects for developing technologies and gaining a holistic and predictive understanding of their system. Using a framework for experimental planning helps bridge theory and data for predictive and mechanistic understanding of biological processes (Lopatkin and Collins, 2020), which has come to the forefront as the field moves away from survey studies (Arkin and Schaffer, 2011; Widder et al., 2016; Prosser and Martiny, 2020). Combined utilization of this framework will overcome current barriers in microbial ecology, allowing for discovery and refinements phases to be iterated leading to a more process-focused predictive outcome.

## A Conceptual Framework for Predictive Microbial Ecology Study Design

**FIGURE 1:**
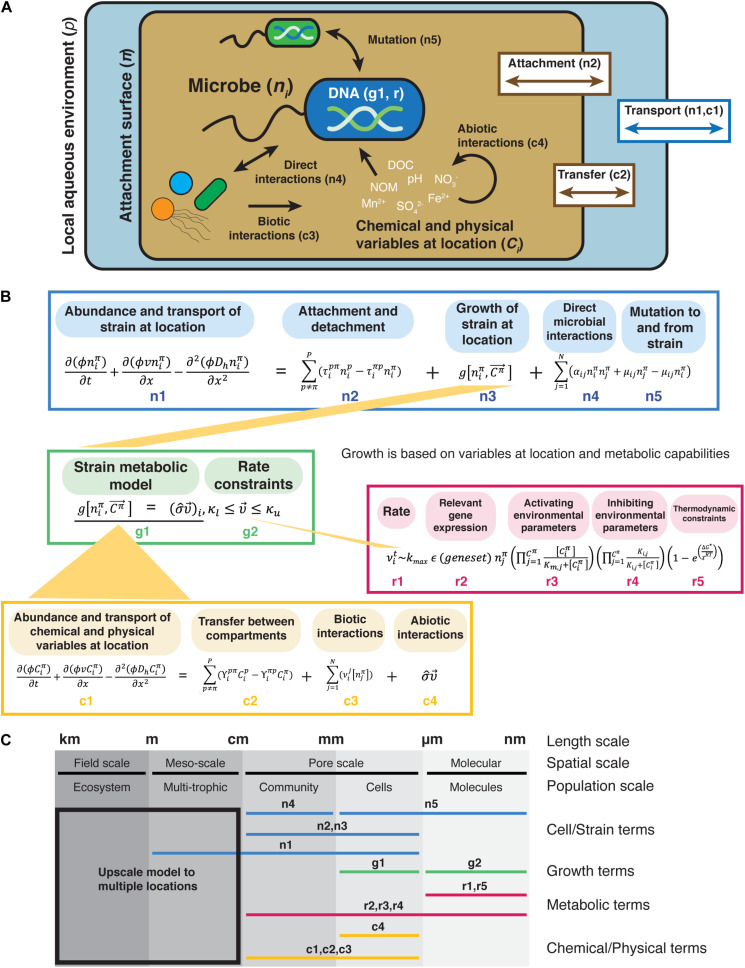
Graphical representation of the Framework for Integrated, Conceptual, and Systematic Microbial Ecology (FICSME). **(A)** Pictorial representation of the framework. **(B)** Conceptual modeling framework. Equations representing potential terms and relationships. **(C)** Scales where the different terms are measured. This framework aims to model the fitness of an organism in a specific environment and spans from the molecular and gene scale to the pore-scale and meso-scale (also referred to as the REV or Darcy scale) and can also be upscaled to the field scale but does not model processes at that scale. The change in abundance of the strain *n*_*i*_ at location π is represented by reactive transport model terms (mass accumulation rate, dispersive/diffusion transport, and advective transport) at the meso-scale (term n1 in **B**), which takes into account porosity (*φ*), abiotic transport (υ), and hydrodynamic dispersion (*D*_*h*_) over time and space. Dispersal is accounted for in terms n1 and n2, but the forces such as water flow rate and rain that might affect dispersal are not explicitly represented here. Physical transport also affects the abundance of strain *n*_*i*_ based on its attachment and detachment from different compartments (e.g., liquid vs. surface), where the transport rate between compartments is *τ* (term n2). The transfer to and from location π is represented by an equation similar to a linear compartmental model. The intrinsic growth of the strain based on its metabolic capabilities under the chemical and physical conditions at the location (term n3). We do not provide a specific equation for growth because here we represent it by the output of a metabolic model (term g1). We use a metabolic model rather than a population growth model (e.g., Monod, Logistic, etc.) because we are representing growth as determined by the chemical and physical conditions (that change over time) and gene content. Biotic factors that affect the abundance of strain *n*_*i*_ are direct biotic interactions (term n4), and mutation to and from the strain, where μ*_*ij*_* is the mutation rate from microbes *n*_*i*_ and *n*_*j*_ (term n5). We represent biotic interactions with term *a*_*ij*_, which is the coefficient representing the strength and sign (positive or negative) of the interaction between microbes *n*_*i*_ and *n*_*j*_. Note that we require this to be a direct, physical interaction rather than a general catchall coefficient that can incorporate indirect (chemical) interactions, such as secretion of antibiotics or other secondary metabolites. These types of indirect interactions are captured in the chemical and metabolic terms. For both, the growth rate of the strain depends on the chemical and physical variables at the location (term c1), which are in turn affected by physical transport between compartments (term c2), biotic transformation of chemicals by microbes (term c3), and abiotic interactions (term c4). The change in abundance of the abundance of chemical and physical variables is also represented by reaction transport terms. For chemical *C*_*i*_, the transport coefficient is γ. Biotic transformation of chemicals is represented by the rate laws for various transformations (υ_*i*_) depending on the microbe that is transforming it. Abiotic reactions of chemicals are represented by a matrix of stoichiometric coefficients for each reaction (σ) and can be thought of as interactions between chemicals, such as oxidation. The intrinsic growth of the strain is represented by a net growth term (term n3). The notation is inspired by constraint-based metabolic models that use flux balance analysis, but it represents anything that affects the growth of strain *n*_*i*_. Here, the constraints (κ) bound the rates v_i_ of the chemical transformations (term g2). The rate v_i_ depends on the enzyme turnover rate, which is determined by the activity of relevant enzymes under Michaelis–Menten enzyme kinetics (term r2), activating and inhibiting environmental parameters (terms r3 and r4), and thermodynamic constraints. Physiological heterogeneity is missing from the growth term (term n3) but could be added into this framework.

To provide a framework for conducting process-focused microbial ecology studies, we have incorporated biological, chemical, and physical processes of a microbial system into a conceptual model that tracks the abundance of a microbial strain over time at a given location based on intrinsic growth, metabolic capabilities, chemicals, and other microorganisms at the site (Figure 1 and Supplementary Table 1). Microbial ecosystem components and processes are given as mathematical representations that can be parameterized through measurement and experimentation that are based on common microbial ecology models (Box 1). Supplementary Table 1 gives detail on the terms and mathematical symbols used. We recognize that this model contains terms and equations that are for niche-based deterministic processes (e.g., species traits and species interactions) and not stochastic processes (Zhou et al., 2013, 2014; Zhou and Ning, 2017), but this framework provides a starting point. The framework for Integrated, Conceptual, and Systematic Microbial Ecology (FICSME) as portrayed in Figure 1 represents processes deterministically and with continuous variables, whereas certain processes may be better and necessarily represented stochastically and discretely. We encourage adding in the processes relevant for the hypotheses under study. This flexibility allows the user to adapt the framework based on the ecosystem components and processes under study and is a key feature of this framework. With this framework, we are not suggesting that there is one universal way to ask microbial ecology questions; we are proposing a focus on incorporating ecological processes to help the field move beyond correlative studies to those that lead to mechanistic understanding of systems (Prosser, 2020; Prosser and Martiny, 2020). This focus helps scientists confront what is necessary in both measurement type and experimental design to observe and parameterize models incorporating such processes. Using this framework forces the researcher to develop a model of their system or choose the components and processes governing interactions between the selected components.

One goal of this framework is to help researchers span appropriate spatio-temporal scales to construct predictive models and experiments from the gene to the ecosystem level. Key to predicting behavior and controlling microbial communities is linking system components and processes (e.g., species interactions, selection, dispersal, metabolic activity, and physiological state) across relevant scales. Equations inspired by relevant types of models are included in the framework at these different scales (Figures 1B,C), such as metabolic models at the molecular and cellular scales, species interactions at the community/pore scale, and reactive transport models at the ecosystem scale (Succurro and Ebenhöh, 2018). Some parts of the framework require fieldwork, such as measuring the chemical and physical variables, while other parts require lab work, such as analysis of gene content and protein function for metabolic modeling. While focused research efforts are necessary to parameterize parts of the framework, we posit that it is essential to keep the whole in mind to help build a holistic view of the system under study and ultimately improve the predictive findings of individual studies.

## Approach to Applying the Framework for Integrated, Conceptual, and Systematic Microbial Ecology

To achieve predictive understanding of microbial communities in relation to ecological processes from genes to the ecosystem level, the FICSME can guide experimental design for a single study or long-term study of a site or system by exposing knowledge gaps and indicating causal factors. The FICSME congregates several simultaneous continuums within the experimental cycle of prediction and testing: experiments and processes can occur in the field or in the laboratory, from the nanometer to kilometer scale, and have dynamics from milliseconds to decades. The FICSME provides a framework to determine (1) important variables and processes of interest driving the chains of causation in target phenotype presentation, (2) what can be measured directly versus what can be inferred using current technology and existing data, and (3) how to collect and integrate data that account for different data types, sampling resolution in time and space, replicate structure, and model training, testing, and validation.

**FIGURE 2:**
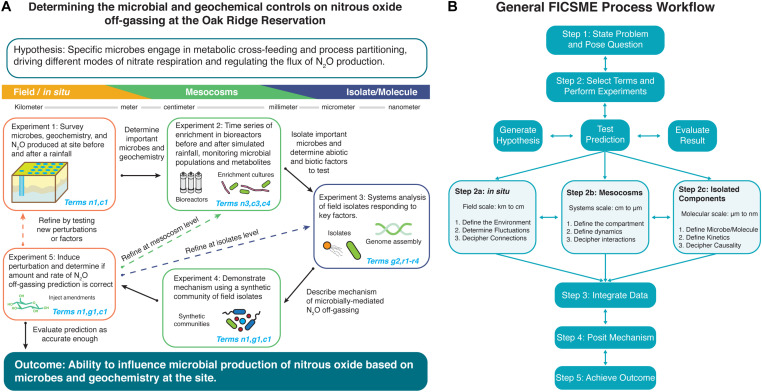
Example of using iterative operations of the Framework for Integrated, Conceptual, and Systematic Microbial Ecology (FICSME) to study nitrous oxide off-gassing. **(A)** Example experimental cycle and **(B)** overall FICSME process diagram. **(A)** We exemplify the process of applying FICSME with an open question in microbial ecology about determining the biogeochemical controls on nitrous oxide off-gassing from nitrate-contaminated sediments. The figure depicts one experimental cycle consisting of a research question or problem, hypothesis, series of five experiments across three scales, integration of data into the model, evaluation of results, opportunity to iterate, or move to outcome. For further details, please see the Tutorial in the Supplementary Material, which includes Supplementary Figure 1 and Supplementary Tables 2, 3. **(B)** Applying the FICSME follows the same guiding principles as the scientific method but incorporates consideration of the FICSME terms at every step. First, the researcher determines what problem they want to study and poses a research question. Then, the researcher will state a specific testable hypothesis, as the FICSME can be used iteratively to address multiple hypotheses and processes that may constitute a larger overarching research question (Step 1). Second, the researcher selects the FICSME terms that are needed to test their hypothesis; this may include removing irrelevant terms from the FICSME or adding terms from other models as appropriate. Then the researcher performs a literature review and checks databases for existing results and data that may satisfy a selected term. The researcher should then populate the FICSME selected terms with these data and identify the knowledge gaps. Next, the researcher will design experiments to fill the identified knowledge gaps and populate the corresponding terms in the FICSME. Each experiment follows the general flow of stating an experimental hypothesis, testing, predicting, and evaluating the result (Step 2). The experiment can be conducted at field scale or *in situ* (Step 2a); at the mesocosm level, which can occur in the field or in the laboratory (Step 2b); or at the isolated molecules level in the laboratory (Step 2c). The FICSME workflow can start at any of these levels and can iterate from one level to any other level (horizontal double arrows). Within each level of experimentation, there are three categories of experiments that can be performed, again in any order, and all might not be required to obtain resolution sufficient for the research question. The three categories are (1) survey or identification and quantification (Steps 2a.1, 2b.1, and 2c.1), (2) dynamics and kinetics (Steps 2a.2, 2b.2, and 2c.2), and (3) interactions and connections (Steps 2a.3, 2b.3, and 2c.3) and are defined for each level of analysis in the figure. Third, the data are collected and the results of individual experiments are evaluated, the data are integrated across scales and techniques, and the total findings are populated into the FICSME (Step 3). Fourth, the collective understanding is used to pose a mechanism giving rise to the target phenotype (Step 4). The mechanism should be tested by performing an experiment from Step 2. This will likely require several iterative cycles to refine the model and prediction. Once the mechanism accurately predicts the system well enough, then the researcher can stop; or fifth, use the quantitative results from the FICSME workflow to intervene in the system to induce the outcome that solves the initial problem identified at the beginning (Step 5).

Since the FICSME is first proposed in this perspective, there are no existing examples that apply this conceptual framework, but we provide case studies that have incorporated aspects of the present framework (Box 3). Herein, we abstractly describe an iterative approach to apply the FICSME (Figure 2B) and then become more concrete by providing a proposed subsurface microbial ecology approach example of nitrous oxide off-gassing (Figure 2A, Supplementary Tutorial, Supplementary Figure 1, and Supplementary Tables 2, 3). Concurrently, we illustrate microbial ecology-specific issues addressed by the FICSME. We emphasize that the FICSME is designed to be generalizable for microbial ecology in any environment, spanning marine sediments to the human gut microbiome to industrial fermentations.

### Define the Microbial Ecology and Research Question

A central challenge in microbial ecology is that some aspects of rigorous, quantitative experimental design and methodology are simply inaccessible such as true replication, absolute abundance, perturbation of measurements on the system, and true time or space series. Using the FICSME in the experimental design helps conceptualize and parameterize complex open environmental systems. Initiating experimental design with the FICSME follows the same approach as the scientific method but applies the framework in each step (Figure 2B). First, define the overarching question of interest including the problem and solution (**Step 1**). Next, a testable hypothesis is established that connects the problem to achieve the outcome. Ideally answering the hypothesis will provide a mechanistic link between observed phenotype, genotype, and environmental factors (see Box 2 for examples). The researcher will then select FICSME terms to be populated and the level of resolution necessary to answer the research question (**Step 2**). Existing data are populated into these selected FICSME terms, and knowledge gaps are identified. The entire FICSME is not meant to be fully parameterized in a single study but can be through multiple studies. The FICSME can be modified to represent the processes and components of interest as necessary.

**Box 2. Questions that support the need for predictive biology and modeling approaches.**

Microbial Ecology Questions

• What are the key processes (abiotic or biotic) driving a particular phenomenon and which organisms are responsible for driving this process?
• How constrained are the organisms responsible for specific processes? Which species are active at the study site and what are the physicochemical conditions? What chemical and physical elements are present, and which abiotic process are active?
• Is there evidence of adaptation and persistence of microbial communities at the site? Is there a persistent core community present at the location, and is it seasonable or trending over time? What are the relative strengths of drift and selection?
• What are the element fluxes per unit area per time due to microbial activity or abiotic reactions over the next N years?
• How can we intervene in a system without affecting ecosystem function (e.g., reduce gases, improve plant productivity, and bioremediation)?
• Are mechanisms governing chemical, biological, and physical phenotypes comparable at sites that are geochemically similar but are geographically distinct?

Mechanistic Modeling Questions

• What is the minimum amount of data and replicates that needs to be measured to achieve the necessary statistical predictive power to answer the question under study? Are the required number of samples feasible taking into account cost, access, and processing?
• How are measurements transformed into actual predictions? And what measurements and modeling capabilities are required to make these predictions?

Example Systems for Application of FICSME

Microbial communities have a profound impact on human health, crop health, and industrial productivity; and, as such, a predictive knowledge of their response to perturbations is vital medically and economically. Situations where a predictive understanding of microbial community function is needed include but are not limited to:

• the gut microbiome and its impacts on human health and disease;
• the fermentation yields of industrial microbes for yogurt, cheese, bread, beer, biofuels, bioplastics, and more;
• the productivity of waste degradation and wastewater treatment facilities;
• improvement of microbes applied in bioremediation; and
• the microbial processes driving biogeochemical cycles and thereby climate change.

### Generate Data Using the Appropriate Experimental System(s)

For generating data, we have categorized experiments into three groups based on the scale and location of the analysis spanning field, laboratory, macroscale, and molecular approaches. At each of these scales, it is necessary to (1) define the environment or compartment(s) under study, (2) take relevant measurements over time to understand fluctuations or kinetics, and (3) determine connections and interactions between system parts. A researcher may enter the experimental cycle at any stage of the continuum. Lack of ability to populate terms indicates areas for technology development in new types of measurements and computational methods, especially at the systems level (Otwell et al., 2018).

Although we emphasize developing experiments with mechanisms in mind, survey studies and statistical modeling can be extremely useful in the discovery phase of a project to help provide focus. Surveys can provide information about the field-relevant ranges of geochemical data, species profiles, and gene expression information. Surveys are also useful if it is difficult to isolate microbial species of interest or to recreate the system conditions in the laboratory (e.g., soil structure or kilometer-scale gradients). Properly designed survey studies that keep downstream statistical analyses in mind can point to the important elements of a system, whether it be biological, chemical, or geophysical, for mechanistic studies.

#### *In situ* Experiments (Step 2a)

Often, a starting point in the continuum will be to conduct discovery-based *in situ* studies. Primary motivations for conducting these studies include when general distinguishing information on the study system is needed; often, this consists of observational surveys. Additionally, *in situ* studies are the primary way for studying microbial dark matter microorganisms that have yet to be cultivated in the laboratory. These studies do not need to be a starting point in using the FICSME and can be used to validate and test hypotheses generated from laboratory studies.

Typically, *in situ* experiments are conducted at the field or ecosystem level. Field- or ecosystem-level experiments act on the largest scale but with the lowest resolution to populate FICSME terms for biotic abundance, transport, transfer and growth (n1, n2, and n3), and the comparable abiotic terms for chemical composition, concentration, and transfer (c1 and c2). The environmental snapshots of natural phenomena detail the necessary context of the overall phenotype that other levels of experiments must be able to predict in order to achieve real-world outcomes. After terms are selected, **Step 2a.1** entails defining the environment by investigating field site geologic zone composition and boundary conditions. This includes determining the compartments, the components (abiotic members) and constituents (biotic members) of each compartment, the respective concentrations or abundances of components, and the functional potential of the location. The study of Smith et al. (2015) is an example of a recent biogeochemical survey of a shallow subsurface groundwater environment, and studies continue to improve and become more comprehensive and mechanistic (Palumbo et al., 2004; Fields et al., 2006; Smith et al., 2015).

For **Step 2a.2**, determining fluctuations of intensity and periodicity involves using the same approach as Step 2a.1, but in time-series studies to gather information on how the composition and abundance of microbial communities and chemicals change over time (both short and long term) (Hwang et al., 2009; Hug et al., 2015). Finally, for **Step 2a.3**, deciphering connections answers how, where, and the rate chemicals and microorganisms are being transferred and transported between compartments and around the location as a whole, encompassing terms like residence time, drift, and dispersion as exemplified in push–pull tests of a uranium-contaminated karst site (Paradis et al., 2018).

There are numerous challenges and barriers associated with *in situ* studies (Griebler and Lueders, 2009; Rocha et al., 2016; Smith et al., 2018; Zelaya et al., 2019). These include the difficulty to sample at sufficient spatio-temporal resolution and the fact that when working in the natural environment external variables/forces cannot be controlled or separated out, which often results in confounding variables. Once hypotheses have been generated or when a greater degree of control is needed to get at more focused process-driven outcomes moving to a different experimental system, i.e., mesocosm- or isolate-level studies are appropriate.

#### Mesocosm Experimental Systems (Step 2b)

While field scale measurements provide insights as to which biotic and abiotic components are most critical to observed function, mesocosms are useful where the distinguishing features of two environment types are specific functional taxonomic groups and particular environmental variations. Mesocosm experiments are then designed for the desired measurements that cannot be taken *in situ*. While the desire is to always match reality and perform experiments with the system in its native state, not all laboratory consortia experiments are precisely motivated by the field but instead test for activities and interactions that could potentially occur. In order to refine observations and hypotheses to generate a more focused and process-driven outcome, mesocosms are a natural step to gain more precise control over an environment; increase observability, direct comparisons, and measurement accessibility; and allow replicate structures that would be impossible in the field.

Mesocosm-scale experiments employ a similar approach to Step 2a but occur in controlled lab or field settings mimicking field conditions where two or more microorganisms, in synthetic or enrichment communities, are grown in mesocosms, microcosms, or various bioreactors. These laboratory systems scale (genome, proteome, and metabolome) reductionist methods reveal the impacts of perturbations on a particular phenotype and serve to populate FICSME biotic terms around attachment and detachment (n2), strain metabolism (g1 and n3), direct microbial interactions (n4) and mutation rates (n5), and abiotic terms for transfer (c2), biotransformations of chemicals (c3), and abiotic chemical reactions (c4). These experiments reveal mechanisms of microbial community assembly, stability, and resilience.

Experiments at the mesocosm scale require first defining the biotic and abiotic members of compartments (**Step 2b.1**) such as in a stratified sediment column where geochemistry and microbial community composition can be measured across sections (Engelbrektson et al., 2014, 2018; Handley et al., 2015) or between bulk soil and the rhizosphere (Starr et al., 2019; Blazewicz et al., 2020; Nuccio et al., 2020). These measurements in time series with replicates or repeated samplings following induced perturbations determine the dynamics of the system by documenting changes in concentrations and abundance, but also understanding transfer or exchange between two compartments (**Step 2b.2**) (Hu et al., 2005; Sher et al., 2020). Once these data have been gathered, deciphering interactions answers how the biotic or abiotic reactions of microbes and chemicals, directly or indirectly, alter the activity or phenotype of the system (**Step 2b.3**) [see programs like Web of Microbes for indirect exometabolite interactions that link mutualists through (Kosina et al., 2018) nutrient competition; EcoFab for rhizosphere direct and indirect interactions toward quorum sensing, predation, or niche exclusion] (Zhalnina et al., 2018; Zengler et al., 2019). Subsequent microbial enrichment cultivation studies can be used to further infer interactions between chemical components and microbial members of low-complexity enrichment cultures (Wawrik et al., 2005; Goldfarb et al., 2011; Carlson et al., 2015b, 2020; Datta et al., 2016; Flynn et al., 2017; Justice et al., 2017; Goldford et al., 2018; Rivett and Bell, 2018; Wu et al., 2018).

Although less complex than *in situ* studies, mesocosm experimental systems have challenges in achieving a high-enough level of mimicry of the native environment (Otwell et al., 2018). This includes obtaining appropriate isolates, identifying the right community members to represent the process of interest, and finding the right growth conditions to as accurately as possible simulate environmental conditions (e.g., soil structure or holistic ecosystem components like microeukaryotes, fungi, or viruses) (Rosenberg et al., 2009; Henkes et al., 2018). Likewise, determining the microorganisms responsible within a community for producing a certain metabolite or facilitating a certain interaction behavior is also challenging without highly targeted methods like stable isotope probing (Blazewicz et al., 2020; Nuccio et al., 2020). Therefore, moving to smaller scales and higher levels of resolution to interrogate the genes responsible for interactions or processes at the isolate scale can provide the needed understanding not obtainable at the mesocosm level.

#### Isolated Microorganisms or Molecular Experimental Systems (Step 2c)

Experiments on single isolated microorganisms or specific molecules provide the most control and the highest resolution to populate FICSME matrix terms that deal with abiotic and biotic reaction rate constants (r1–r5, g2, and c4) and amount and activity of individual catalysts such as molecules, enzymes, isolates, or model microorganisms. The subject of these experiments is often the critical organisms and environmental parameters determined from survey, *in situ*, or mesocosm experiments. These types of experiments include physiological or bioinformatics-based characterization of isolates, linking genes to function or specific molecules, and characterization of produced metabolites or proteins. Advances in laboratory automation facilitate controlled experimental studies to measure how complex multi-dimensional gradients impact microbial interactions and complex microbiomes (Carlson et al., 2017, 2019, 2020).

Experiments at this scale first require defining the microbe or molecule for **Step 2c.1** (Cheng et al., 2013; Liu et al., 2018; Price et al., 2018; Xue et al., 2020). Determining kinetics of these components encompasses measurements of rates of change in genomic sequences, enzyme activities, and the chemical reactions occurring in the environment (**Step 2c.2**). As strains evolve, the genotype of the system is altered. In response to changes in genotype or environmental conditions, high-resolution time-series experiments pinpoint molecular changes to individual genes, proteins, and metabolites as in adaptive laboratory evolution studies (Stoeva et al., 2020; Wu et al., 2020). Likewise, enzyme activity assays parameterize the kinetic constraints of microbial respirations that drive field phenotype presentation as in many studies on respiratory enzymes (Martens-Habbena et al., 2009; Stahl and de la Torre, 2012; Youngblut et al., 2016; Mehta-Kolte et al., 2019; Straka et al., 2019). The system phenotype is also controlled by abiotic reactions, which are determined by measuring their kinetic rate constants as in studies on the reactivity between nitrogen and sulfur species and iron minerals (Hansen et al., 1996; Carlson et al., 2012; Flynn et al., 2014; Grabb et al., 2017). To decipher causation for **Step 2c.3**, molecular reductionist methods answer how a particular molecule or microorganism is acting to alter phenotype at high resolution (Carlson et al., 2015a; Thorgersen et al., 2015; Vaccaro et al., 2016; Price et al., 2018; Ge et al., 2020). For example, pooled mutant fitness assays can be used to help determine gene functions of an organism (Price et al., 2018).

Although experiments focused on isolates or specific molecules provide the most control, there are still many challenges for execution and field relevance (Palková, 2004; de Boer, 2017; Zhalnina et al., 2018; Barreto et al., 2020; Cornforth et al., 2020). As previously mentioned, it may be difficult to isolate some species because they require syntrophic partners or because growth conditions are unknown (Stewart, 2012). Some organisms are also not amenable to current genetic manipulation methods. Despite major recent advances, bioinformatics challenges still include genome assembly and gene function annotation. Technology and methods development are also needed for characterizing and studying unknown organic matter and metabolites. Findings at the isolates and molecules scale can turn into hypotheses to be tested at the other scales to demonstrate if the phenomena happen under more realistic conditions.

### Data Integration and Iteration for Integrated, Conceptual, and Systematic Microbial Ecology

The typical iteration of models and experiments applies here (create a model, test with experimental data, and refine model based on results), but the FICSME encourages iterations that include gathering data from different scales to improve accuracy of prediction. This may mean coordinated work across multiple studies and expertise across disciplines (Box 3). Iteration may also mean studying other processes within the same scale that affects the focus of your study. Having an iterative cycle across scales encourages having initial studies to help define boundaries of the study system and determine variable importance—this helps with reducing the number of variables to test.

**BOX 3. Example case studies.**

Determining Field-Relevant Factors Influencing Competition Between *Rhodoferax* and *Geobacter* (Single Study)

An example of a successful study bridging scales from genes to the ecosystem level was conducted by Zhuang et al. (2011) who investigated the conditions favoring the presence of either *Rhodoferax* or *Geobacter*, the dominant species at the Rifle, CO contaminated subsurface research site. *Geobacter* can reduce U(VI) to an insoluble form while *Rhodoferax* cannot; thus, the abundance of these two species can impact the bioremediation capabilities of uranium at the site (**Step 1**). The authors investigated how acetate and ammonium could impact competition between *Rhodoferax* and *Geobacter*. In this study, field measurements of nutrient flux and other relevant parameters were estimated based on results from previous studies at the site. These estimates were used as input into flux balance analysis (FBA) models of the two species (**Step 2**). Additionally, the authors developed a modeling framework to integrate genome-scale metabolic models into a community metabolic model (**Step 3**). Simulation results suggested that depending on the acetate and ammonium concentrations, *Geobacter* could outcompete *Rhodoferax* by resource competition (**Step 4**), which was determined by simulating acetate injections (**Step 5**). Zhuang et al. (2011) suggested that to improve mechanistic understanding of the microbial and chemical dynamics at the site, the impact of other species interactions on *in situ* bioremediation could be investigated, as well as incorporating of reactive transport models with the FBA models to help study the effect of ecosystem-level events (Scheibe et al., 2009) **(potential iteration of FICSME)**.

Large Team Investigation of an Anthropogenically Contaminated Terrestrial Subsurface Site (Multiple Integrated Studies)

The U.S. Department of Energy Science Focus Area ENIGMA seeks to understand the biogeochemical processes in the Oak Ridge Reservation (ORR), a Manhattan project uranium enrichment site. ORR is a superfund site due to leaching of hazardous waste from unlined retention ponds (Kornegay et al., 1994; Brooks, 2001; Revil et al., 2013; Thorgersen et al., 2019). We hypothesize that dissimilatory nitrate reduction (DNR) is the primary process toward remediating the ORR site and immobilizing toxic metals, namely, uranium. Specifically, we are interested in discovering the constraints on DNR in the subsurface of ORR with mechanistic understanding of the ecological phenomena and system components affecting this process from the gene level to the ecosystem level (**Step 1**).

To link processes and factors from the gene scale to the ecosystem scale, ENIGMA has multiple studies at the field scale (**Step 2a**), mesocosm scale (**Step 2b**), and molecular/species level (**Step 2c**). To facilitate these studies, ENIGMA has major research thrusts aimed at field surveys (Smith et al., 2015; Paradis et al., 2018; Zelaya et al., 2019; Moon et al., 2020); laboratory and bioreactor studies of isolates (Vaccaro et al., 2016; Price et al., 2018; Carlson et al., 2019; Thorgersen et al., 2019); syncoms, enrichments, and improved isolation methods (Wu et al., 2018, 2019); genetic tool development (Liu et al., 2018; Price et al., 2018; Mutalik et al., 2019); and bioinformatics analyses and tools (Price and Arkin, 2019; Lui et al., 2020; Price et al., 2020).

At the field scale, ENIGMA has conducted surveys (**Step 2a.1**) to characterize the biotic and abiotic components of the subsurface using 16S amplicon and shotgun metagenomics sequencing to characterize the microbial communities (Smith et al., 2015; Zelaya et al., 2019; Tian et al., 2020) and biogeochemical measurements for the abiotic factors (Smith et al., 2015; Ge et al., 2020; Moon et al., 2020; Wu et al., 2020). Hydrology and topology studies of ORR indicate that there are significant groundwater flow rates influenced by frequent rain events (Watson et al., 2005), and preliminary tracer measurements with push–pull tests determined dispersal rates of chemicals like nitrate and their transfer between compartments (Paradis et al., 2018) (**Step 2a.2**). Sampling of sediment and groundwater from pristine and contaminated areas (Zhang et al., 2017) across time scales (Zelaya et al., 2019) confirmed geochemistry influencing highly variable communities (Smith et al., 2015; Hemme et al., 2016; Thompson et al., 2017a; He et al., 2018; Carlson et al., 2019; Thorgersen et al., 2019; Tian et al., 2019) (**Step 2a.3**). These field studies provide an overview of the chemical and microbial landscape at ORR, which suggests that dispersal is a highly impactful ecological process on the microbial communities, and indicate directions for focused studies at the mesocosm and isolate levels.

At the mesocosm level, ENIGMA has conducted enrichment, bioreactor, and synthetic community studies (**Step 2b**). Bioreactor studies found transfer rates between sediment particle attachment or groundwater planktonic compartments (Justice et al., 2017; King et al., 2017; Christensen et al., 2018; Smith et al., 2018; Wilpiszeski et al., 2020) (**Step 2b.2**). Enrichment studies have determined that different carbon sources determine the active DNR pathway and select for specific species (Carlson et al., 2020) (**Step 2b.3**).

ENIGMA has isolated thousands of bacterial and archaeal strains from the ORR site and is exploring the impact of biogeochemistry at field-relevant levels on the survival and function of these isolates (Wu et al., 2018; Carlson et al., 2019; Thorgersen et al., 2019; Wu et al., 2019) (**Step 2c.1**). At the isolate and molecular levels, we have found that major selective pressures also include concentration, heavy metals, nitrate, and low pH, which alter DNR microbial growth (Carlson et al., 2019; Thorgersen et al., 2019). Iron- and aluminum-induced molybdenum removal can inhibit nitrate reduction in the acidic conditions at ORR (Ge et al., 2019) (**Step 2c.3**). Respiration by-product exometabolites (Kosina et al., 2016, 2018) from sulfate-reducing bacteria stimulated DNR to ammonia, while inhibiting other DNR enzymes (Otwell et al., 2021). With the use of isolates under nitrate-reducing conditions, high-throughput gene fitness assays (Vaccaro et al., 2016), global stable isotope metabolomics profiling (Kurczy et al., 2016), and assessing substrate and cofactor requirements of key DNR enzymes (Vuono et al., 2019; Carlson et al., 2020) indicated highly selective controls on DNR pathway usage. Bioavailable molybdenum above a certain concentration is essential to support DNR (Thorgersen et al., 2015; Ge et al., 2019) and regulated through controls on molybdate transporters (Rajeev et al., 2019). Thus, to stimulate DNR activity as a way of returning the site to pristine conditions, the method should be chosen based on the knowledge of the differential responses of DNR pathways enzymes, cofactors, and coenzymes to changing environmental conditions.

Our studies of the environment, microbes, transport, and DNR activity have been synthesized into initial testing of predictions about microbial community responses to perturbations in the ORR subsurface (**Step 3**) (Zhang et al., 2015a,b; Paradis et al., 2016). Based on the knowledge gathered from Steps 1–3 in the ORR subsurface and other DNR communities in uranium-contaminated sites, high nitrate and uranium concentrations co-occur with low pH areas, which causes depletion of bioavailable molybdenum, an essential nitrate reductase cofactor (Thorgersen et al., 2015). In the most contaminated wells, low pH, high uranium, manganese, aluminum, cadmium, cobalt, and nickel are selective pressures that exclude select for resistant microorganisms such as *Rhodanobacter* (Carlson et al., 2019). Consequently, most DNR microbes are excluded or inhibited, except those that tolerate low pH and high concentrations of metals. In addition to donor or acceptor limitations on DNR activity rates, we assert that low pH, by controlling abiotic and biotic interactions, is the dominant constraint on DNR in the subsurface. We predict that amendments to raise the pH and add carbon sources to electron donor limited regions of the site will result in an increase of DNR activity with a corresponding decrease in nitrate contamination and eventual decreased uranium mobility following dynamic succession to more reducing metabolisms (**Step 4**). To test this prediction, we are conducting a Design of Experiments process integrating cross-discipline and scale, field, and laboratory studies (**Step 5**). As part of the iteration process (**Step 5**), we are applying the FICSME to help with coordinating experiments for time series, more precise geochemical measurements and monitoring, new methods for tracking microbial strains through sequencing, standardization of methodology, *in situ* and *ex situ* activity measurements (more precise), ability to count microbes within compartments and maintain sediment and community structure, and development of new bioreactor studies.

For **Step 3** of the FICSME, single studies integrate the multi-scale data into the framework to either predict outcomes (e.g., microbiome composition predicted from geochemistry) or answer their hypotheses to gain mechanistic insights. This permits the quantitative assessment of the accuracy of the model prediction or the resolution of the outcome. Ideally, model predictions at one scale dovetail with other scales and predictions can be tested based on multi-scale knowledge. Based on the results of prediction testing, for **Step 4**, the researcher will formulate a new mechanistic model describing the occurrence and variation of the target phenotype or process. If the model was experimentally and quantitatively validated, the researcher moves on to **Step 5**, if appropriate. If the new model was not accurate enough, the researcher iterates on this experimental cycle to gain sufficient data on parameters (**Steps 2–3**) until a sufficient resolution of understanding is ascertained. Once experiments have validated the model, for **Step 5**, the researcher can perturb the system through amendments or the necessary means determined in **Steps 1–4**, thereby changing the phenotype to provide a solution to the target problem.

### Proposed Use of Framework for Integrated, Conceptual, and Systematic Microbial Ecology to Quantitatively and Mechanistically Predict Flux of N_2_O Off-Gassing

Microbial interaction-driven processes underscore global challenges in health and the environment. We describe a proposed approach using the FICSME to gain predictive understanding of nitrous oxide off-gassing from nitrate-contaminated soils and sediments, a major contributor to climate change (Figure 2A). Existing models may accurately simulate total flux during model calibration [i.e., the Landscape DeNitrification DeComposition (DNDC) model, which predicts N_2_O emissions from agricultural management variables (Molina-Herrera et al., 2016)] but do not include microbial processes and may not perform well in model prediction. What is missing is knowledge of phenomenon-specific microbial community activities characterized *in situ* and across scales. Using the FICSME, researchers can add organismal and molecular resolution mechanisms to these models; doing so pinpoints actionable interventions addressing this global problem. A workflow is described herein and depicted in Figure 2, while a detailed tutorial including Supplementary Figure 1 and Supplementary Tables 2, 3.

Nitrous oxide (N_2_O) off-gassing from nitrate-contaminated soils and sediments is a microbially mediated process that contributes a harmful greenhouse gas to the problem of climate change (Step 1: state the problem). This leads to the overarching research question of “What are the microbial and geochemical controls on nitrous oxide off-gassing from the heavily nitrate contaminated subsurface at the Oak Ridge Reservation?,” which we describe in Box 3 (Step 1: state the question). While N_2_O is produced by microbial metabolisms collectively carrying out complete denitrification, the amount produced and released is controlled by the geochemistry of the site. Through association of geochemical and microbial respiration activity measurements at the same depth from previous field observations, we can hypothesize that specific microbes are engaged in metabolic cross-feeding and process partitioning to drive different modes of nitrate respiration, depending on environmental context (Step 2: generate testable hypothesis).

Since both abiotic and biotic factors appear to govern the amount and rate of N_2_O off-gassing, their respective FICSME terms must be considered to understand and accurately predict the response to a perturbation in the system (Step 2: select the terms). For this research question and hypothesis, we will focus on FICSME terms for membership, abundance, concentration, growth, interactions, and enzyme activity, but not transport or dispersal in this iteration (although they might be determined to be important later). Then, we consider existing knowledge about the processes contributing to N_2_O off-gassing from nitrate-contaminated environments to populate terms and identify knowledge gaps. This yields specific sub-hypotheses about the concurrent contributions of abiotic and biotic factors such as (1) carbon source and electron donor preferences and availability stimulating different microbes and metabolisms; (2) low pH inhibiting NosZ enzyme, which converts N_2_O to nitrogen gas on the denitrification pathway; (3) the availability of molybdenum, an essential cofactor for nitrate reductase enzyme activity to convert nitrate to nitrite; (4) the concentration and oxidation states of iron and manganese driving chemodenitrification; and (5) the production of sulfide gas via sulfate-reducing organisms with the ability to shift nitrate respiration mode from denitrification to dissimilatory nitrate reduction to ammonia (DNRA).

The FICSME can be used to iteratively incorporate all hypotheses and concomitant processes and factors. For this example of a proposed plan, one hypothesis would be tested at a time by changing the factors or perturbations tested at each stage of the proposed experimental cycle and then iterating as necessary. Experiment 1 follows Steps 2a.1 and 2a.2 seeking to populate terms n1 and c1 by sampling the subsurface and groundwater to monitor the changes in composition of the microbial community, concentrations of geochemical parameters, and amount of N_2_O off-gassing before and after a rainfall event, which alters the geochemistry, nutrient availability, and community membership. With the responsive microbes and geochemistries identified, they are selected for enrichment and factor testing in Experiment 2. Experiment 2 follows Steps 2b.1 and 2b.2 seeking to populate terms n3, c3, and c4 by growing the enriched field communities in replicate bioreactors that attempt to mimic the geochemistry and sediment structure from the corresponding depth in the subsurface. Perturbations are applied to the bioreactors that attempt to simulate environmental processes of interest like rainfall events. The changes to the community and chemistry are monitored with higher-resolution techniques, true and more replicates, and finer time-series samplings that assess the response of individual organisms, genes, proteins, and metabolites. The key responsive microbes and chemicals are then isolated. Experiment 3 follows Steps 2c.1–3 seeking to populate terms r1–r4 by studying in depth isolated microorganisms, enzymes, metabolites, or abiotic factors. This can include in-depth characterization of regulation, toxicity mechanisms, nitrogen metabolism, gene function, and enzyme activity, all assayed in a variety of field-mimicking conditions and over time that establish the boundaries of the behavior of each molecule and microbe. The amassed knowledge from molecular reductionist studies will lead to proposing a mechanism that describes the chain of causality between the flux in biotic and abiotic factors during a rainfall event that leads to the observable phenotype in changes in the amount of N_2_O off-gassing. Experiment 4 follows Step 2b.3 seeking to populate terms n1, g1, and c1 at the mesocosm level by populating the same bioreactor system in Experiment 2 with a synthetic community of the isolates from Experiment 3 that collectively will simulate the environment and phenotype by carrying out complete denitrification. A perturbation is induced quantitatively to test the mechanism proposed at the conclusion of Experiment 3 and measured over time. If the synthetic community validates the mechanism at this mesocosm level, then the prediction is tested back in the field. Experiment 5 follows Steps 2a.2 and 2a.3 seeking to populate terms n1, g1, and c1 by introducing a perturbation into the field-testing site and monitoring the results of the prediction based on the determined mechanism.

After the experimental cycle is completed, the data are integrated, and the results of the prediction testing are assessed for accuracy to a resolution matching the needs of the research question defined in Step 1. If the prediction based on the determined mechanism is accurate enough, then the researcher can move to implementing the prescribed intervention to produce the desired outcome or system phenotype. For this example, the ultimate outcome would be to add an amendment of a microbe or a chemical that would regulate nitrous oxide off-gassing at the desired rate and amount. If, however, the prediction is not accurate or other processes need to be included, then the researcher iterates on the process by going back to any of the previous steps or proposing new experiments as necessary within the confines of the FICSME.

## Discussion: Future Challenges for Microbial Ecology

To overcome critical limitations in the transition of microbial ecology to a quantitative and predictive discipline, there is a need for integrating results across scales and the many concomitant processes in an ecosystem and formal Design of Experiments calculations, a statistics-based method for determining causal relationships between factors and outcomes and guiding appropriate sample selection, to balance sufficiently powered surveys to guide mechanistic experiments. Integration across scales and the inclusion of the microbial component have yielded the benefits of precise knowledge on the behavior of a microorganism or enzyme under a certain set of conditions (Gao et al., 2020). This type of model-informed sampling will ultimately strengthen our ability to understand the effect of biological and chemical processes within an environment such that we can intervene and achieve a precise desired outcome for microbial systems.

Depending on the research question, different mathematical models and different parts of the FICSME will be relevant and whether single or multiple studies are needed. We encourage users to add relevant models or phenomena. For example, physiological heterogeneity of cells is not represented. In regard to experimental planning, a single “campaign” might start by planning out the series of different sorts of models that will allow building a more mechanistic one; e.g., a control-treatment model that identifies taxa most separated by environmental variables might allow to focus attention on measurement of the variation of these in more mechanistic experiments.

Parameterizing the FICSME from multiple studies in different systems and from different groups quickly runs into challenges related to metrology, metadata collection, and data standardization (Navas-Molina et al., 2017). Data from different studies may not be compatible because of the methods used, so documentation of metadata (e.g., sample type) and other methods (e.g., DNA extraction can influence which species are sequenced) are becoming especially important. Efforts such as the Earth microbiome project (Gilbert et al., 2014; Thompson et al., 2017b), DOE Systems Biology KnowledgeBase (Arkin et al., 2018), and the National Microbiome Data Collaborative (Wood-Charlson et al., 2020) are attempting to do so. Data quality standards and efforts such as the FAIR data principles (Findability, Accessibility, Interoperability, Reusability) are also critical so that as we build models we propagate error appropriately (Wilkinson et al., 2016). Increasing the molecular information through large-scale programs can help provide databases and distribute effort to collect hard-to-obtain data. For models to be generalizable and benefit from other studies, data need to be FAIR, computational tools to be open and accessible, and analyses to be reusable and reproducible. Initiatives like KBase are building platforms for data, analytical tools and models together in one place, all adhering to FAIR principles (Arkin et al., 2018). Continued discussion and thought are needed for how to make data interoperable and how data from different analytical pipelines and different measurement modalities (e.g., amplicon and metagenomic inferred taxonomic abundance) can be combined together. Using systems like KBase, subsurface insights, ESS-DIVE, Web of Microbes (Kosina et al., 2018), METLIN MS^2^ (Xue et al., 2020), and NSF/USGS NEON can help.

Our work in developing the FICSME to achieve mechanistic understanding has pointed to these challenges and needs:

(1) Expertise to conduct multiple complex measurement modalities and develop models across field and laboratory scales.
(2) Gathering the correct data categories for model parameterization.
(3) Data analysis may be computationally intensive.
(4) Limited ability to gather enough replicates for statistics or impossibility of measuring all variables.
(5) Incompatible data types, or lack of mathematical method for combining results of different types, especially with different resolutions and dynamics.
(6) Inter-lab inconsistencies in procedures or need for standardized methods, data collection, ontologies, and standard operating procedures across labs.

We suggest the following to overcome these challenges in pursuit of quantitative, mechanistic, predictive microbial ecology:

(1) Multidisciplinary team science approach that spans the expertise needed to integrate data and methodologies across scales.
(2) Use of formal Design of Experiments to help bridge scales, design experiments that point to mechanism for observed ecological phenomena, and coordinate across multiple studies. This should be linked with rigorous reporting and annotation of protocols for measurement and data analysis using standardized ontologies.
(3) Use of machine learning methods such as neural networks to help find patterns in data to direct focused experiments, based on identifying the most important variables with predictive power.
(4) Iterative experimental design from field to lab, survey to mechanism, prediction/hypothesis to testing *in situ*.
(5) Improved data sharing and recording of metadata programs and efforts, including tracking the provenance of samples and data.

## Data Availability Statement

The original contributions presented in the study are included in the article/Supplementary Material, further inquiries can be directed to the corresponding author/s.

## Author Contributions

LL, EM, and HS contributed equally in the conception, writing, editing, and figure making of this manuscript. HC, NB, and FvN contributed portions of the text and editing. DS, MF, and JZ were involved in model ideation and editing of the manuscript. PA and TH were involved in manuscript conception. AA was primarily responsible for model creation and provided feedback, and editing of manuscript throughout the process. All authors contributed to the article and approved the submitted version.

## Conflict of Interest

The authors declare that the research was conducted in the absence of any commercial or financial relationships that could be construed as a potential conflict of interest.

## References

[B1] Arkin A. P. Cottingham R. Henry C. S. Harris N. L. Stevens R. L. Maslov S. (2018). KBase: the united states department of energy systems biology knowledgebase. *Nat. Biotechnol.* 36 566–569. 29979655 10.1038/nbt.4163 PMC6870991

[B2] Arkin A. P. Schaffer D. V. (2011). Network news: innovations in 21st century systems biology. *Cell* 144 844–849. 10.1016/j.cell.2011.03.008 21414475

[B3] Barreto H. C. Cordeiro T. N. Henriques A. O. Gordo I. (2020). Rampant loss of social traits during domestication of a *Bacillus subtilis* natural isolate. *Sci. Rep.* 10:18886. 10.1038/s41598-020-76017-1 PMC7642357 33144634

[B4] Blazewicz S. J. Hungate B. A. Koch B. J. Nuccio E. E. Morrissey E. Brodie E. L. (2020). Taxon-specific microbial growth and mortality patterns reveal distinct temporal population responses to rewetting in a California grassland soil. *ISME J.* 14 1520–1532. 10.1038/s41396-020-0617-3 32203117 PMC7242442

[B5] Brooks S. C. (2001). *Waste Characteristics of the Former S-3 Ponds and Outline of Uranium Chemistry Relevant to NABIR Field Research Center studies.* Oak Ridge, TN: NABIR Field Research Center.

[B6] Carlson H. Deutschbauer A. Coates J. (2017). Microbial metal resistance and metabolism across dynamic landscapes: high-throughput environmental microbiology. *F1000Res.* 6:1026. 10.12688/f1000research.10986.1 28721211 PMC5497819

[B7] Carlson H. K. Clark I. C. Melnyk R. A. Coates J. D. (2012). Toward a mechanistic understanding of anaerobic nitrate-dependent iron oxidation: balancing electron uptake and detoxification. *Front. Microbiol.* 3:57. 10.3389/fmicb.2012.00057 22363331 PMC3282478

[B8] Carlson H. K. Kuehl J. V. Hazra A. B. Justice N. B. Stoeva M. K. Sczesnak A. (2015a). Mechanisms of direct inhibition of the respiratory sulfate-reduction pathway by (per)chlorate and nitrate. *ISME J.* 9 1295–1305. 10.1038/ismej.2014.216 25405978 PMC4438318

[B9] Carlson H. K. Stoeva M. K. Justice N. B. Sczesnak A. Mullan M. R. Mosqueda L. A. (2015b). Monofluorophosphate is a selective inhibitor of respiratory sulfate-reducing microorganisms. *Environ. Sci. Technol.* 49 3727–3736. 10.1021/es505843z 25698072

[B10] Carlson H. K. Lui L. M. Price M. N. Kazakov A. E. Carr A. V. Kuehl J. V. (2020). Selective carbon sources influence the end products of microbial nitrate respiration. *ISME J.* 14 2034–2045. 10.1038/s41396-020-0666-7 32372050 PMC7368043

[B11] Carlson H. K. Price M. N. Callaghan M. Aaring A. Chakraborty R. Liu H. (2019). The selective pressures on the microbial community in a metal-contaminated aquifer. *ISME J.* 13 937–949. 10.1038/s41396-018-0328-1 30523276 PMC6461962

[B12] Cheng X. Hiras J. Deng K. Bowen B. Simmons B. A. Adams P. D. (2013). High throughput nanostructure-initiator mass spectrometry screening of microbial growth conditions for maximal β-glucosidase production. *Front. Microbiol.* 4:365. 10.3389/fmicb.2013.00365 eCollection 2013 24367356 PMC3854461

[B13] Christensen G. A. Moon J. Veach A. M. Mosher J. J. Wymore A. M. van Nostrand J. D. (2018). Use of in-field bioreactors demonstrate groundwater filtration influences planktonic bacterial community assembly, but not biofilm composition. *PLoS One* 13:e0194663. 10.1371/journal.pone.0194663 29558522 PMC5860781

[B14] Cornforth D. M. Diggle F. L. Melvin J. A. Bomberger J. M. Whiteley M. (2020). Quantitative framework for model evaluation in microbiology research using *Pseudomonas aeruginosa* and cystic fibrosis infection as a test case. *mBio* 11:e03042-19. 10.1128/mbio.03042-19 31937646 PMC6960289

[B15] Datta M. S. Sliwerska E. Gore J. Polz M. F. Cordero O. X. (2016). Microbial interactions lead to rapid micro-scale successions on model marine particles. *Nat. Commun.* 7:11965. 10.1038/ncomms11965 PMC4915023 27311813

[B16] de Boer W. (2017). Upscaling of fungal–bacterial interactions: from the lab to the field. *Curr. Opin. Microbiol.* 37 35–41. 10.1016/j.mib.2017.03.007 28437664

[B17] Engelbrektson A. Briseno V. Liu Y. Figueroa I. (2018). Mitigating sulfidogenesis with simultaneous perchlorate and nitrate treatments. *Front. Microbiol.* 9:2305. 10.3389/fmicb.2018.02305 30337913 PMC6180152

[B18] Engelbrektson A. Hubbard C. G. Tom L. M. Boussina A. Jin Y. T. Wong H. (2014). Inhibition of microbial sulfate reduction in a flow-through column system by (per)chlorate treatment. *Front. Microbiol.* 5:315. 10.3389/fmicb.2014.00315 25071731 PMC4092371

[B19] Fields M. W. Bagwell C. E. Carroll S. L. Yan T. Liu X. Watson D. B. (2006). Phylogenetic and functional biomakers as indicators of bacterial community responses to mixed-waste contamination. *Environ. Sci. Technol.* 40 2601–2607. 10.1021/es051748q 16683598

[B20] Flynn T. M. Koval J. C. Greenwald S. M. Owens S. M. Kemner K. M. Antonopoulos D. A. (2017). Parallelized, aerobic, single carbon-source enrichments from different natural environments contain divergent microbial communities. *Front. Microbiol.* 8:2321. 10.3389/fmicb.2017.02321 29234312 PMC5712364

[B21] Flynn T. M. O’Loughlin E. J. Mishra B. DiChristina T. J. Kemner K. M. (2014). Sulfur-mediated electron shuttling during bacterial iron reduction. *Science* 344 1039–1042. 10.1126/science.1252066 24789972

[B22] Gao Q. Wang G. Xue K. Yang Y. Xie J. Yu H. (2020). Stimulation of soil respiration by elevated CO2 is enhanced under nitrogen limitation in a decade-long grassland study. *PNAS* 117 33317–33324. 10.1073/pnas.2002780117 33318221 PMC7777058

[B23] Ge X. Thorgersen M. P. Poole F. L. Deutschbauer A. M. Chandonia J.-M. Novichkov P. S. (2020). Characterization of a metal-resistant bacillus strain with a high molybdate affinity ModA from contaminated sediments at the oak ridge reservation. *Front. Microbiol.* 11:2543. 10.3389/fmicb.2020.587127 33193240 PMC7604516

[B24] Ge X. Vaccaro B. J. Thorgersen M. P. Poole F. L. II Majumder E. L. Zane G. M. (2019). Iron- and aluminium-induced depletion of molybdenum in acidic environments impedes the nitrogen cycle. *Environ. Microbiol.* 21 152–163. 10.1111/1462-2920.14435 30289197

[B25] Gilbert J. A. Jansson J. K. Knight R. (2014). The earth microbiome project: successes and aspirations. *BMC Biol.* 12:69. 10.1186/s12915-014-0069-1 25184604 PMC4141107

[B26] Goldfarb K. C. Karaoz U. Hanson C. A. Santee C. A. Bradford M. A. Treseder K. K. (2011). Differential growth responses of soil bacterial taxa to carbon substrates of varying chemical recalcitrance. *Front. Microbiol.* 2:94. 10.3389/fmicb.2011.00094 21833332 PMC3153052

[B27] Goldford J. E. Lu N. Bajić D. Estrela S. Tikhonov M. Sanchez-Gorostiaga A. (2018). Emergent simplicity in microbial community assembly. *Science* 361 469–474. 10.1126/science.aat1168 30072533 PMC6405290

[B28] Grabb K. C. Buchwald C. Hansel C. M. Wankel S. D. (2017). A dual nitrite isotopic investigation of chemodenitrification by mineral-associated Fe(II) and its production of nitrous oxide. *Geochim. Cosmochim. Acta* 196 388–402. 10.1016/j.gca.2016.10.026

[B29] Griebler C. Lueders T. (2009). Microbial biodiversity in groundwater ecosystems. *Freshw. Biol.* 54 649–677. 10.1111/j.1365-2427.2008.02013.x

[B30] Guo X. Feng J. Shi Z. Zhou X. Yuan M. Tao X. (2018). Climate warming leads to divergent succession of grassland microbial communities. *Nat. Clim. Change* 8 813–818. 10.1038/s41558-018-0254-2

[B31] Guo X. Gao Q. Yuan M. Wang G. Zhou X. Feng J. (2020). Gene-informed decomposition model predicts lower soil carbon loss due to persistent microbial adaptation to warming. *Nat Commun* 11:4897. 10.1038/s41467-020-18706-z PMC7524716 32994415

[B32] Handley K. M. Wrighton K. C. Miller C. S. Wilkins M. J. Kantor R. S. Thomas B. C. (2015). Disturbed subsurface microbial communities follow equivalent trajectories despite different structural starting points. *Environ. Microbiol.* 17 622–636. 10.1111/1462-2920.12467 24674078

[B33] Hansen H. C. B. Koch C. B. Nancke-Krogh H. Borggaard O. K. Sørensen J. (1996). Abiotic nitrate reduction to ammonium: key role of green rust. *Environ. Sci. Technol.* 30 2053–2056. 10.1021/es950844w

[B34] He Z. Zhang P. Wu L. Rocha A. M. Tu Q. Shi Z. (2018). Microbial functional gene diversity predicts groundwater contamination and ecosystem functioning. *mBio* 9:e02435-17. 10.1128/mbio.02435-17 29463661 PMC5821090

[B35] Hemme C. L. Green S. J. Rishishwar L. Prakash O. Pettenato A. Chakraborty R. (2016). Lateral gene transfer in a heavy metal-contaminated-groundwater microbial community. *mBio* 7:e02234-15. 10.1128/mBio.02234-15 PMC4817265 27048805

[B36] Henkes G. J. Kandeler E. Marhan S. Scheu S. Bonkowski M. (2018). Interactions of mycorrhiza and protists in the rhizosphere systemically alter microbial community composition, plant shoot-to-root ratio and within-root system nitrogen allocation. *Front. Environ. Sci.* 6:117. 10.3389/fenvs.2018.00117

[B37] Hu S. Wu J. Burkey K. O. Firestone M. K. (2005). Plant and microbial N acquisition under elevated atmospheric CO2 in two mesocosm experiments with annual grasses. *Glob. Change Biol.* 11 213–223. 10.1111/j.1365-2486.2005.00905.x

[B38] Hug L. A. Thomas B. C. Brown C. T. Frischkorn K. R. Williams K. H. Tringe S. G. (2015). Aquifer environment selects for microbial species cohorts in sediment and groundwater. *ISME J.* 9 1846–1856. 10.1038/ismej.2015.2 25647349 PMC4511941

[B39] Hwang C. Wu W. Gentry T. J. Carley J. Corbin G. A. Carroll S. L. (2009). Bacterial community succession during in situ uranium bioremediation: spatial similarities along controlled flow paths. *ISME J.* 3 47–64. 10.1038/ismej.2008.77 18769457

[B40] Justice N. B. Sczesnak A. Hazen T. C. Arkin A. P. (2017). Environmental selection, dispersal, and organism interactions shape community assembly in high-throughput enrichment culturing. *Appl. Environ. Microbiol.* 83: e01253-17. 10.1128/AEM.01253-17 PMC5626985 28778896

[B41] King A. J. Preheim S. P. Bailey K. L. Robeson M. S. II Roy Chowdhury T. Crable B. R. (2017). Temporal dynamics of in-field bioreactor populations reflect the groundwater system and respond predictably to perturbation. *Environ. Sci. Technol.* 51 2879–2889. 10.1021/acs.est.6b04751 28112946

[B42] Kornegay F. West D. McMahon L. Murphy J. Shipe L. Koncinski W. (1994). “Oak ridge reservation annual site environmental report for 1993,” in *ES/ESH-47.* Bethesda, MA: Martin Marietta Energy Systems, Inc.

[B43] Kosina S. M. Danielewicz M. A. Mohammed M. Ray J. Suh Y. Yilmaz S. (2016). Exometabolomics assisted design and validation of synthetic obligate mutualism. *ACS Synth. Biol.* 15, 569–576. 10.1021/acssynbio.5b00236 26885935

[B44] Kosina S. M. Greiner A. M. Lau R. K. Jenkins S. Baran R. Bowen B. P. (2018). Web of microbes (WoM): a curated microbial exometabolomics database for linking chemistry and microbes. *BMC Microbiol.* 18:115. 10.1186/s12866-018-1256-y 30208844 PMC6134592

[B45] Kurczy M. E. Forsberg E. M. Thorgersen M. P. Poole F. L. II Benton H. P. Ivanisevic J. (2016). global isotope metabolomics reveals adaptive strategies for nitrogen assimilation. *ACS Chem. Biol.* 11 1677–1685. 10.1021/acschembio.6b00082 27045776 PMC5730404

[B46] Liu H. Price M. N. Waters R. J. Ray J. Carlson H. K. Lamson J. S. (2018). Magic pools: parallel assessment of transposon delivery vectors in bacteria. *mSystems* 3:e00143-17. 10.1128/mSystems.00143-17 29359196 PMC5768790

[B47] Lopatkin A. J. Collins J. J. (2020). Predictive biology: modelling, understanding and harnessing microbial complexity. *Nat. Rev. Microbiol.* 18 507–520. 10.1038/s41579-020-0372-5 32472051

[B48] Lui L. M. Nielsen T. N. Arkin A. P. (2020). A method for achieving complete microbial genomes and improving bins from metagenomics data. *bioRxiv* [Preprint]. 10.1101/2020.03.05.979740 PMC8172020 33961626

[B49] Maher K. Mayer K. U. (2019). The art of reactive transport building. *Elements* 15:117. 10.2138/gselements.15.2.117

[B50] Martens-Habbena W. Berube P. M. Urakawa H. de la Torre J. R. Stahl D. A. (2009). Ammonia oxidation kinetics determine niche separation of nitrifying Archaea and Bacteria. *Nature* 461 976–979. 10.1038/nature08465 19794413

[B51] Mehta-Kolte M. G. Stoeva M. K. Mehra A. Redford S. A. Youngblut M. D. Zane G. (2019). Adaptation of *Desulfovibrio alaskensis* G20 to perchlorate, a specific inhibitor of sulfate reduction. *Environ. Microbiol.* 21 1395–1406. 30807684 10.1111/1462-2920.14570

[B52] Meile C. Scheibe T. D. (2019). Reactive transport modeling of microbial dynamics. *Elements* 15 111–116. 10.2138/gselements.15.2.111

[B53] Molina-Herrera S. Haas E. Klatt S. Kraus D. Augustin J. Magliulo V. (2016). A modeling study on mitigation of N2O emissions and NO3 leaching at different agricultural sites across Europe using LandscapeDNDC. *Sci. Total Environ.* 553 128–140. 10.1016/j.scitotenv.2015.12.099 26909705

[B54] Moon J.-W. Paradis C. J. Joyner D. C. von Netzer F. Majumder E. L. Dixon E. R. (2020). Characterization of subsurface media from locations up- and down-gradient of a uranium-contaminated aquifer. *Chemosphere* 255:126951. 10.1016/j.chemosphere.2020.126951 32417512

[B55] Mutalik V. K. Novichkov P. S. Price M. N. Owens T. K. Callaghan M. Carim S. (2019). Dual-barcoded shotgun expression library sequencing for high-throughput characterization of functional traits in bacteria. *Nat. Commun.* 10:308. 10.1038/s41467-018-08177-8 PMC6338753 30659179

[B56] Navas-Molina J. A. Hyde E. R. Sanders J. G. Knight R. (2017). The microbiome and big data. *Curr. Opin. Syst. Biol.* 4 92–96. 10.1016/j.coisb.2017.07.003 PMC10019530 36937228

[B57] Ning D. Yuan M. Wu L. Zhang Y. Guo X. Zhou X. (2020). A quantitative framework reveals ecological drivers of grassland microbial community assembly in response to warming. *Nat. Commun.* 11:4717. 10.1038/s41467-020-18560-z 32948774 PMC7501310

[B58] Nordstrom D. K. Kirk Nordstrom D. (2012). Models, validation, and applied geochemistry: issues in science, communication, and philosophy. *Appl. Geochem.* 27 1899–1919. 10.1016/j.apgeochem.2012.07.007

[B59] Nuccio E. E. Starr E. Karaoz U. Brodie E. L. Zhou J. Tringe S. G. (2020). Niche differentiation is spatially and temporally regulated in the rhizosphere. *ISME J.* 14 999–1014. 10.1038/s41396-019-0582-x 31953507 PMC7082339

[B60] Otwell A. E. Carr A. V. Majumder E. L.-W. Ruiz M. K. Wilpiszeski R. L. Hoang L. T. (2021). Sulfur metabolites play key system-level roles in modulating denitrification. *mSystems* 6, e01025–20. 10.1128/mSystems.01025-20 33563788 PMC7883540

[B61] Otwell A. E. López García, de Lomana A. Gibbons S. M. Orellana M. V. Baliga N. S. (2018). Systems biology approaches towards predictive microbial ecology. *Environ. Microbiol.* 20 4197–4209. 10.1111/1462-2920.14378 30106224

[B62] Palková Z. (2004). Multicellular microorganisms: laboratory versus nature. *EMBO Rep.* 5 470–476. 10.1038/sj.embor.7400145 15184977 PMC1299056

[B63] Palumbo A. V. Schryver J. C. Fields M. W. Bagwell C. E. Zhou J.-Z. Yan T. (2004). Coupling of functional gene diversity and geochemical data from environmental samples. *Appl. Environ. Microbiol.* 70 6525–6534. 10.1128/aem.70.11.6525-6534.2004 15528515 PMC525260

[B64] Paradis C. J. Jagadamma S. Watson D. B. McKay L. D. Hazen T. C. Park M. (2016). In situ mobility of uranium in the presence of nitrate following sulfate-reducing conditions. *J. Contam. Hydrol.* 187 55–64. 10.1016/j.jconhyd.2016.02.002 26897652

[B65] Paradis C. J. McKay L. D. Perfect E. Istok J. D. Hazen T. C. (2018). Push-pull tests for estimating effective porosity: expanded analytical solution and in situ application. *Hydrogeol. J.* 26 381–393. 10.1007/s10040-017-1672-3

[B66] Price M. N. Arkin A. P. (2019). Curated BLAST for genomes. *mSystems* 4:e00072-19. 10.1128/mSystems.00072-19 30944879 PMC6435814

[B67] Price M. N. Deutschbauer A. M. Arkin A. P. (2020). GapMind: automated annotation of amino acid biosynthesis. *mSystems* 5:e00291-20. 10.1128/mSystems.00291-20 32576650 PMC7311316

[B68] Price M. N. Wetmore K. M. Waters R. J. Callaghan M. Ray J. Liu H. (2018). Mutant phenotypes for thousands of bacterial genes of unknown function. *Nature* 557 503–509. 10.1038/s41586-018-0124-0 29769716

[B69] Prosser J. I. (2020). Putting science back into microbial ecology: a question of approach. *Philos. Trans. R. Soc. Lond. B Biol. Sci.* 375:20190240. 10.1098/rstb.2019.0240 32200745 PMC7133526

[B70] Prosser J. I. Martiny J. B. H. (2020). Conceptual challenges in microbial community ecology. *Philos. Trans. R. Soc. Lond. B Biol. Sci.* 375:20190241. 10.1098/rstb.2019.0241 32200750 PMC7133534

[B71] Rajeev L. Garber M. E. Zane G. M. Price M. N. Dubchak I. Wall J. D. (2019). A new family of transcriptional regulators of tungstoenzymes and molybdate/tungstate transport. *Environ. Microbiol.* 21 784–799. 30536693 10.1111/1462-2920.14500

[B72] Revil A. Skold M. Karaoulis M. Schmutz M. Hubbard S. S. Mehlhorn T. L. (2013). Hydrogeophysical investigations of the former S-3 ponds contaminant plumes, Oak Ridge Integrated Field Research Challenge site, Tennessee. *Geophysics* 78 EN29–EN41.

[B73] Rivett D. W. Bell T. (2018). Abundance determines the functional role of bacterial phylotypes in complex communities. *Na. Microbiol.* 3 767–772. 10.1038/s41564-018-0180-0 29915204 PMC6065991

[B74] Rocha A. M. Yuan Q. Close D. M. O’Dell K. B. Fortney J. L. Wu J. (2016). Rapid detection of microbial cell abundance in aquatic systems. *Biosens. Bioelectron.* 85 915–923. 10.1016/j.bios.2016.05.098 27315516

[B75] Rosenberg K. Bertaux J. Krome K. Hartmann A. Scheu S. Bonkowski M. (2009). Soil amoebae rapidly change bacterial community composition in the rhizosphere of Arabidopsis thaliana. *ISME J.* 3 675–684. 10.1038/ismej.2009.11 19242534

[B76] Scheibe T. D. Mahadevan R. Fang Y. Garg S. Long P. E. Lovley D. R. (2009). Coupling a genome-scale metabolic model with a reactive transport model to describe in situ uranium bioremediation. *Microb. Biotechnol.* 2 274–286. 10.1111/j.1751-7915.2009.00087.x 21261921 PMC3815847

[B77] Sher Y. Baker N. R. Herman D. Fossum C. Hale L. Zhang X. (2020). Microbial extracellular polysaccharide production and aggregate stability controlled by switchgrass (*Panicum virgatum*) root biomass and soil water potential. *Soil Biol. Biochem.* 143:107742. 10.1016/j.soilbio.2020.107742

[B78] Smith H. J. Zelaya A. J. De León K. B. Chakraborty R. Elias D. A. Hazen T. C. (2018). Impact of hydrologic boundaries on microbial planktonic and biofilm communities in shallow terrestrial subsurface environments. *FEMS Microbiol. Ecol.* 94:fiy191. 10.1093/femsec/fiy191 30265315 PMC6192502

[B79] Smith M. B. Rocha A. M. Smillie C. S. Olesen S. W. Paradis C. Wu L. (2015). Natural bacterial communities serve as quantitative geochemical biosensors. *mBio* 6:e00326-15. 10.1128/mBio.00326-15 PMC4436078 25968645

[B80] Stahl D. A. de la Torre J. R. (2012). Physiology and diversity of ammonia-oxidizing archaea. *Annu. Rev. Microbiol.* 66 83–101. 10.1146/annurev-micro-092611-150128 22994489

[B81] Starr E. P. Nuccio E. E. Pett-Ridge J. Banfield J. F. Firestone M. K. (2019). Metatranscriptomic reconstruction reveals RNA viruses with the potential to shape carbon cycling in soil. *Proc. Natl. Acad. Sci. U.S.A.* 116 25900–25908. 10.1073/pnas.1908291116 31772013 PMC6926006

[B82] Stewart E. J. (2012). Growing unculturable bacteria. *J. Bacteriol.* 194 4151–4160. 10.1128/jb.00345-12 22661685 PMC3416243

[B83] Stoeva M. K. Kuehl J. Kazakov A. E. Wang O. Rushton-Green R. Coates J. D. (2020). Anion transport as a target of adaption to perchlorate in sulfate-reducing communities. *ISME J.* 14 450–462. 10.1038/s41396-019-0540-7 31659234 PMC6976614

[B84] Stokes C. Arkin A. (2007). “Modeling and network organization,” in *Systems Biology. Springer, Dordrecht. A. Modeling and Network Organization*, eds Cassman M. Arkin A. Doyle F. Katagiri F. Lauffenburger D. Stokes C. (Berlin: Springer), 47–81. 10.1007/978-1-4020-5468-6_4

[B85] Straka L. L. Meinhardt K. A. Bollmann A. Stahl D. A. Winkler M.-K. (2019). Affinity informs environmental cooperation between ammonia-oxidizing archaea (AOA) and anaerobic ammonia-oxidizing (Anammox) bacteria. *ISME J.* 13 1997–2004. 10.1038/s41396-019-0408-x 30936420 PMC6775968

[B86] Succurro A. Ebenhöh O. (2018). Review and perspective on mathematical modeling of microbial ecosystems. *Biochem. Soc. Trans.* 46 403–412. 10.1042/bst20170265 29540507 PMC5906705

[B87] Thompson A. W. Turkarslan S. Arens C. E. López García de Lomana A. Raman A. V. Stahl D. A. (2017a). Robustness of a model microbial community emerges from population structure among single cells of a clonal population. *Environ. Microbiol.* 19 3059–3069. 10.1111/1462-2920.13764 28419704

[B88] Thompson L. R. The Earth Microbiome Project Consortium Sanders J. G. McDonald D. Amir A. Ladau J. (2017b). A communal catalogue reveals Earth’s multiscale microbial diversity. *Nature* 551 457–463. 29088705 10.1038/nature24621 PMC6192678

[B89] Thorgersen M. P. Ge X. Poole F. L. II Price M. N. Arkin A. P. Adams M. W. W. (2019). Nitrate-utilizing microorganisms resistant to multiple metals from the Heavily Contaminated Oak Ridge Reservation. *Appl. Environ. Microbiol.* 85:e00896-19. 10.1128/AEM.00896-19 31253673 PMC6696972

[B90] Thorgersen M. P. Lancaster W. A. Vaccaro B. J. Poole F. L. Rocha A. M. Mehlhorn T. (2015). Molybdenum availability is key to nitrate removal in contaminated groundwater environments. *Appl. Environ. Microbiol.* 81 4976–4983. 10.1128/aem.00917-15 25979890 PMC4495186

[B91] Tian T. Kang J. W. Kang A. Lee T. S. (2019). Redirecting metabolic flux via combinatorial multiplex CRISPRi-Mediated repression for isopentenol production in *Escherichia coli*. *ACS Synth. Biol.* 8 391–402. 10.1021/acssynbio.8b00429 30681833

[B92] Tian R. Ning D. He Z. Zhang P. Spencer S. J. Gao S. (2020). Small and mighty: adaptation of superphylum Patescibacteria to groundwater environment drives their genome simplicity. *Microbiome* 8, 51. 10.1186/s40168-020-00825-w 32252814 PMC7137472

[B93] Vaccaro B. J. Thorgersen M. P. Lancaster W. A. Price M. N. Wetmore K. M. Poole F. L. II (2016). Determining roles of accessory genes in denitrification by mutant fitness analyses. *Appl. Environ. Microbiol.* 82 51–61. 10.1128/aem.02602-15 26452555 PMC4702625

[B94] van Mourik T. Bühl M. Gaigeot M. P. (2014). Density functional theory across chemistry, physics and biology. *Philos. Trans. Ser. A Math. Phys. Eng.Scie.* 372:20120488. 10.1098/rsta.2012.0488 PMC3928866 24516181

[B95] Venable R. M. Kramer A. Pastor R. W. (2019). molecular dynamics simulations of membrane permeability. *Chem. Rev.* 119 5954–5997. 30747524 10.1021/acs.chemrev.8b00486 PMC6506413

[B96] Vuono D. C. Read R. W. Hemp J. Sullivan B. W. Arnone J. A. III Neveux I. (2019). Resource concentration modulates the fate of dissimilated nitrogen in a dual-pathway Actinobacterium. *Front. Microbiol.* 10:3. 10.3389/fmicb.2019.00003 30723459 PMC6349771

[B97] Wagner M. (2015). Microbiology: conductive consortia. *Nature* 526 513–514. 10.1038/526513a 26490616

[B98] Watson D. Doll W. Jeffrey Gamey T. Sheehan J. Jardine P. (2005), Plume and lithologic profiling with surface resistivity and seismic tomography. *Groundwater* 43, 169–177. 10.1111/j.1745-6584.2005.0017.x 15819938

[B99] Wawrik B. Kerkhof L. Kukor J. Zylstra G. (2005). Effect of different carbon sources on community composition of bacterial enrichments from soil. *Appl. Environ. Microbiol.* 71 6776–6783. 10.1128/aem.71.11.6776-6783.2005 16269709 PMC1287611

[B100] Widder S. Allen R. J. Pfeiffer T. Curtis T. P. Wiuf C. Sloan W. T. (2016). Challenges in microbial ecology: building predictive understanding of community function and dynamics. *ISME J.* 10 2557–2568. 27022995 10.1038/ismej.2016.45 PMC5113837

[B101] Wilkinson M. D. Dumontier M. Aalbersberg I. J. J. Appleton G. Axton M. Baak A. (2016). The FAIR guiding principles for scientific data management and stewardship. *Sci. Data* 3:160018. 10.1038/sdata.2016.18 PMC4792175 26978244

[B102] Wilpiszeski R. L. Gionfriddo C. M. Wymore A. M. Moon J.-W. Lowe K. A. Podar M. (2020). In-field bioreactors demonstrate dynamic shifts in microbial communities in response to geochemical perturbations. *PLoS One* 15:e0232437. 10.1371/journal.pone.0232437 32986713 PMC7521895

[B103] Wood-Charlson E. M. Anubhav, Auberry D. Blanco H. Borkum M. I. Corilo Y. E. (2020). The national microbiome data collaborative: enabling microbiome science. *Nat. Rev. Microbiol.* 18 313–314. 32350400 10.1038/s41579-020-0377-0

[B104] Wu B. Liu F. Zhou A. Li J. Shu L. Kempher M. L. (2020). Experimental evolution reveals nitrate tolerance mechanisms in *Desulfovibrio vulgaris*. *ISME J.* 14 2862–2876. 10.1038/s41396-020-00753-5 32934357 PMC7784701

[B105] Wu X. Spencer S. Alm E. J. Voriskova J. Chakraborty R. (2019). Capturing the diversity of subsurface microbiota–choice of carbon source for microcosm enrichment and isolation of groundwater bacteria. *bioRxiv* [Preprint]. 10.1101/517854

[B106] Wu X. Wu L. Liu Y. Zhang P. Li Q. Zhou J. (2018). Microbial interactions with dissolved organic matter drive carbon dynamics and community succession. *Front. Microbiol.* 9:1234. 10.3389/fmicb.2018.01234 29937762 PMC6002664

[B107] Xue J. Guijas C. Benton H. P. Warth B. Siuzdak G. (2020). METLIN MS molecular standards database: a broad chemical and biological resource. *Nat. Methods* 17 953–954. 10.1038/s41592-020-0942-5 32839599 PMC8802982

[B108] Youngblut M. D. Tsai C.-L. Clark I. C. Carlson H. K. Maglaqui A. P. Gau-Pan P. S. (2016). perchlorate reductase is distinguished by active site aromatic gate residues. *J. Biol. Chem.* 291 9190–9202. 10.1074/jbc.m116.714618 26940877 PMC4861485

[B109] Zelaya A. J. Parker A. E. Bailey K. L. Zhang P. Van Nostrand J. Ning D. (2019). High spatiotemporal variability of bacterial diversity over short time scales with unique hydrochemical associations within a shallow aquifer. *Water Res.* 164:114917. 10.1016/j.watres.2019.114917 31387058

[B110] Zengler K. Hofmockel K. Baliga N. S. Behie S. W. Bernstein H. C. Brown J. B. (2019). EcoFABs: advancing microbiome science through standardized fabricated ecosystems. *Nat. Methods* 16 567–571. 31227812 10.1038/s41592-019-0465-0 PMC6733021

[B111] Zhalnina K. Zengler K. Newman D. Northen T. R. (2018). Need for laboratory ecosystems to unravel the structures and functions of soil microbial communities mediated by chemistry. *mBio* 9:e01175-18. 10.1128/mBio.01175-18 PMC6050955 30018110

[B112] Zhang P. He Z. Van Nostrand J. D. Qin Y. Deng Y. Wu L. (2017). Dynamic succession of groundwater sulfate-reducing communities during prolonged reduction of uranium in a contaminated aquifer. *Environ. Sci. Technol.* 51 3609–3620. 10.1021/acs.est.6b02980 28300407

[B113] Zhang P. Van Nostrand J. D. He Z. Chakraborty R. Deng Y. Curtis D. (2015a). A slow-release substrate stimulates groundwater microbial communities for long-term in situ Cr(VI) reduction. *Environ. Sci. Technol.* 49 12922–12931. 10.1021/acs.est.5b00024 25835088

[B114] Zhang P. Wu W.-M. Van Nostrand J. D. Deng Y. He Z. Gihring T. (2015b). Dynamic succession of groundwater functional microbial communities in response to emulsified vegetable oil amendment during sustained in situ U (VI) reduction. *Appl. Environ. Microbiol.* 81 4164–4172. 10.1128/aem.00043-15 25862231 PMC4524159

[B115] Zhou J. (2009). Predictive microbial ecology. *Microb. Biotechnol.* 2 154–156. 10.1111/j.1751-7915.2009.00090_21.x 21261909 PMC3815835

[B116] Zhou J. Deng Y. Zhang P. Xue K. Liang Y. Van Nostrand J. D. (2014). Stochasticity, succession, and environmental perturbations in a fluidic ecosystem. *Proc. Natl. Acad. Sci. U.S.A.* 111 E836–E845. 10.1073/pnas.1324044111 24550501 PMC3948316

[B117] Zhou J. Liu W. Deng Y. Jiang Y.-H. Xue K. He Z. (2013). Stochastic assembly leads to alternative communities with distinct functions in a bioreactor microbial community. *mBio* 4 584–512. 10.1128/mbio.00584-12 23462114 PMC3585448

[B118] Zhou J. Ning D. (2017). Stochastic community assembly: does it matter in microbial ecology? *Microbiol. Mol. Biol. Rev.* 81:e00002-17. 10.1128/MMBR.00002-17 29021219 PMC5706748

[B119] Zhuang K. Izallalen M. Mouser P. Richter H. Risso C. Mahadevan R. (2011). Genome-scale dynamic modeling of the competition between Rhodoferax and Geobacter in anoxic subsurface environments. *ISME J.* 5 305–316. 10.1038/ismej.2010.117 20668487 PMC3105697

