# Supplementary material for: Mechanism Across Scales: A Holistic Modeling Framework Integrating Laboratory and Field Studies for Microbial Ecology
Source: Front Microbiol. 2021 Mar 24;12:642422. doi: 10.3389/fmicb.2021.642422 (PMC8024649; doi:10.3389/fmicb.2021.642422)
Supplement: Supplementary file 1 [file Data_Sheet_1.docx]

Supplementary Material

**Supplementary Table 1**. List and Definitions of Individual Terms and Operators found in the Framework for Integrated, Conceptual, and Systematic Microbial Ecology

| **Term** | **Meaning/Definition** | **Assumptions/Notes**  **(where applicable)** | **Equation segments** | **Measurement Scale** |
| --- | --- | --- | --- | --- |
| n | Abundance, number of catalysts | n_i_ number/concentration of a particular species, *i* | n1-5, g1, r2, c3 | μm |
| N | Number of different types or strains of microbes in the model |  | n4 | km |
| C | Environmental parameters including chemical concentrations, porosity, osmolarity, temperature, moisture, etc. | Abiotic variables that may be changing over time due to inputs and biology | n3, g1, r3, r4, c1, c2 | nm-km |
| *i* | One microbial strain, one chemical, one reaction or one interaction |  | all | μm |
| *j* | Another microbial strain, chemical, reaction or interaction |  | all | μm |
| P | Number of separable compartments (attached, sediment pore, main groundwater flow) | Sum shows transfer from each compartment to any other compartment | n2 | mm- km |
| π (pi, lc) | Compartment/location |  | all | mm-m |
| p or ρ (rho, lc) | Compartment/location | A different compartment than π | n2, c2 | mm-m |
| τ (tau, lc) | Transport/transfer rate of microbes between compartment | e.g. transition from attached to planktonic & vice versa | n2 | μm-km |
| ϒ (upsilon, uc) | Transport/transfer rate of chemicals between compartments | Like tau but for chemicals, *e.g.* polysaccharide being attached to sediment or free floating | c2 | μm-km |
| D_h_ | Hydrodynamic dispersion |  | n1, c1 | μm-km |
| φ (phi, lc) | Porosity |  | n1, c1 | nm-m |
| x | physical space, horizontal distance across site | x, y, z coordinate plane | n1, c1 | nm-km |
| t | Time |  | n1, r1 |  |
| g | Specific growth rate of strain n_i_ given the environmental parameters in the compartment |  | n3, g1 | μm-cm |
| ν (nu, lc) | Rate laws for various chemical transformations | Biotic | r1 | nm |
| υ (upsilon, lc) | Rates of abiotic transformations (like nu but abiotic) |  | g2, c4 | nm |
| σ (sigma, lc) | stoichiometric matrix for reactions, e.g. a flux-balance model | Abiotic; essentially a mass-conservation matrix for cellular metabolism | g1, c4 | nm |
| κ (kappa, lc) | Constraints put on rates in a flux-balance model |  | g2 |  |
| α_ij_ (alpha, lc) | Direct species interactions term between species *i* and *j* (Lotka-Volterra like) | Unlike L-V, this model requires that these be "touching" reactions *e.g.*  type-VI secretion | n4 | nm-mm |
| μ (mu, lc) | Mutation, mutation rates | Rate at which species n_j_ can mutate in species n_i_ for example | n5 | nm-μm |
| *gene set* | all of the genes considered in the model |  | r2 | nm-km |
| ε (epsilon, lc) | metabolic enzyme expression level/concentration of each gene in the gene set | An enzyme might be composed of a number of polypeptides. Therefore, its concentration is dependent on the expression of multiple genes (gene set). | r2 | nm-km |
| k_max_ | Enzyme turnover rate | vmax= k_cat*[E_total] | r1 | nm |
| k_m_ | Michaelis-Menten constant (substrate affinity) | K_M_i_j is the Michaelis constant for activation of the i^th^ reaction (regulatorily) by variable Cj | r3 | nm |
| k_i_ | Inhibition constant for enzymes | K_I_i_j is the inhibition constant for the same i^th^ reaction | r4 | nm |
| ΔG | Gibbs Free Energy for the reaction | Used to determine directionality of reaction | r5 | nm |
| R | Universal Gas Constant |  | r5 | nm |
| T | Temperature (in Kelvin) |  | r5 | nm-km |
| **Operators** | **Meaning/Definition** | **Assumptions/Notes** |  |  |
| Forward arrow | Vector |  |  |  |
| Hat ^ | Hamiltonian vector, estimator, differential equation **Matrix** |  |  |  |
| δ/δt (delta, lc) | Rate (partial derivative with respect to time) | Appears to be part of a multivariate differential equation |  |  |
| δ/δx (delta, lc) | 1^st^ derivative (partial derivative with respect to distance) | Appears to be part of a multivariate differential equation |  |  |
| δ^2^/δx^2^ (delta, lc) | 2^nd^ derivative (partial derivative with respect to distance) | Appears to be part of a multivariate differential equation |  |  |
| Σ (sigma, uc) | Summation | Repeated addition |  |  |
| Π (pi, uc) | Product Notation | Repeated multiplication |  |  |
| ~ | Approximately equals |  |  |  |
| e | Base of the natural log |  |  |  |
| ( ) | Groups variables such that they are operated on together |  |  |  |
| [ ] | Functional notation |  |  |  |
| lc, uc | lower case, uppercase |  |  |  |

Tutorial

**Tutorial for Example of Using Iterative Operations of FICSME to Study Nitrous Oxide Flux Emissions from Terrestrial Sedimentary Microbiomes.**

Below we seek to outline step by step how to use FICSME to complete one experimental cycle. This tutorial demonstrates the integration of the equation-based model with the overall process using a pertinent knowledge gap in microbial ecology as an example. The tutorial outlines one experimental cycle for one hypothesis but describes the points at which iteration may occur to either refine the model or to add additional processes and hypotheses and how the decision to iterate should be made. This example is not meant to be comprehensive nor complete, but rather instructive on how to approach using FICSME.

**Step 1:**

State the Problem:

Contamination at the Oak Ridge Reservation (ORR) consists of a low pH, high nitrate, high toxic metal plume that is leaching into the Bear Creek Valley (see Box 3, paragraph 1). Nitrous oxide (N_2_O) off-gassing from nitrate contaminated soils and sediments is a both an abiotic and microbial-mediated process that contributes a harmful greenhouse gas to the problem of climate change.

Pose Overarching Research Question:

This leads to the overarching research question of “What are the microbial and geochemical controls on nitrous oxide fluxes from the heavily nitrate contaminated subsurface at the Oak Ridge Reservation?”

**Step 2:**

Generate Specific Testable Hypothesis:

While biotic N_2_O is produced in part by microbial metabolisms collectively carrying out denitrification, the rates and amount produced and released is controlled by the geochemistry of the site. Geochemical regulations on this microbial process would include carbon source, nutrient and cofactor availability, physical community structure in the subsurface, and the ability of gasses to diffuse through the subsurface. Abiotic processes can also contribute to nitrous oxide fluxes, as some minerals and inorganic ions can catalyze chemodenitrification. Based on the previous work by the ENIGMA project described in Box 3 and summarized in the tables below, which revealed the association of geochemical and microbial respiration activity measurements at the same depth, we can hypothesize that *resident sedimentary microbes are engaged in metabolic cross-feeding and process partitioning to catalyze different steps of nitrate respiration and that they compete with abiotic processes depending on environmental context.* The first experimental cycle presented here targets denitrification to nitrous oxide by classical nitrate reducing bacteria. Upon completing this first cycle, we expect that the model would not be accurate enough because of the likely contribution of the many other groups of microbes that could be producing nitrous oxide, as well as a significant fraction from abiotic reactions that drive chemodenitrification.

Select FICSME terms needed to test hypothesis:

Since both abiotic and biotic factors appear to govern N_2_O fluxes, their respective FICSME terms must be considered to understand and accurately predict the response to a perturbation in the system. For this research question and hypothesis, we will focus on FICSME terms for a focused subset of biotic and abiotic processes, taxa and biogeochemical components (parts of n1, c1). This means focusing chemical and enzymatically driven denitrification reactions that would appear in c3, c4 and g2; microbial growth that appears in n3 and g1, and enzyme activity (g2, r1-r4), as represented in Supplementary Figure 1, Supplementary Tables 2 and 3. We chose these parameters because we first need to understand the regulation and mechanisms of denitrification in one compartment of the site. Once we can accurately predict the amount of nitrous oxide off-gassing from one compartment, then the model can be expanded to include more compartments and the connections between them to be able to predict amounts of nitrous oxide flux measured across the site. So, we will not consider the terms transport (part of n1, c1), dispersal (part of n1, c1), attachment (n2), transfer (c2), direct microbial interactions (n4), mutations (n5) or thermodynamics (r5) in this iteration (Supplementary Figure 1). We did not select, for example, direct interaction terms (n4) because we are assuming that the amount of direct interactions is negligible due to distance between microbes in sediment particles. Also, while transport and dispersal are important to overall flux at the whole site, we are predicting for the sake of this tutorial that these terms are not important to the mechanism of nitrous oxide off-gassing in one compartment. After completing this iteration, we might find that our model predicts all compartments from the site well without incorporating these terms, or we may learn that factors like dispersion are dominant drivers of the site even within one compartment. The iterative nature of FICSME will allow us to test these additional factors by adding back in those terms or other modules that describe that factor better and refine the model accordingly.


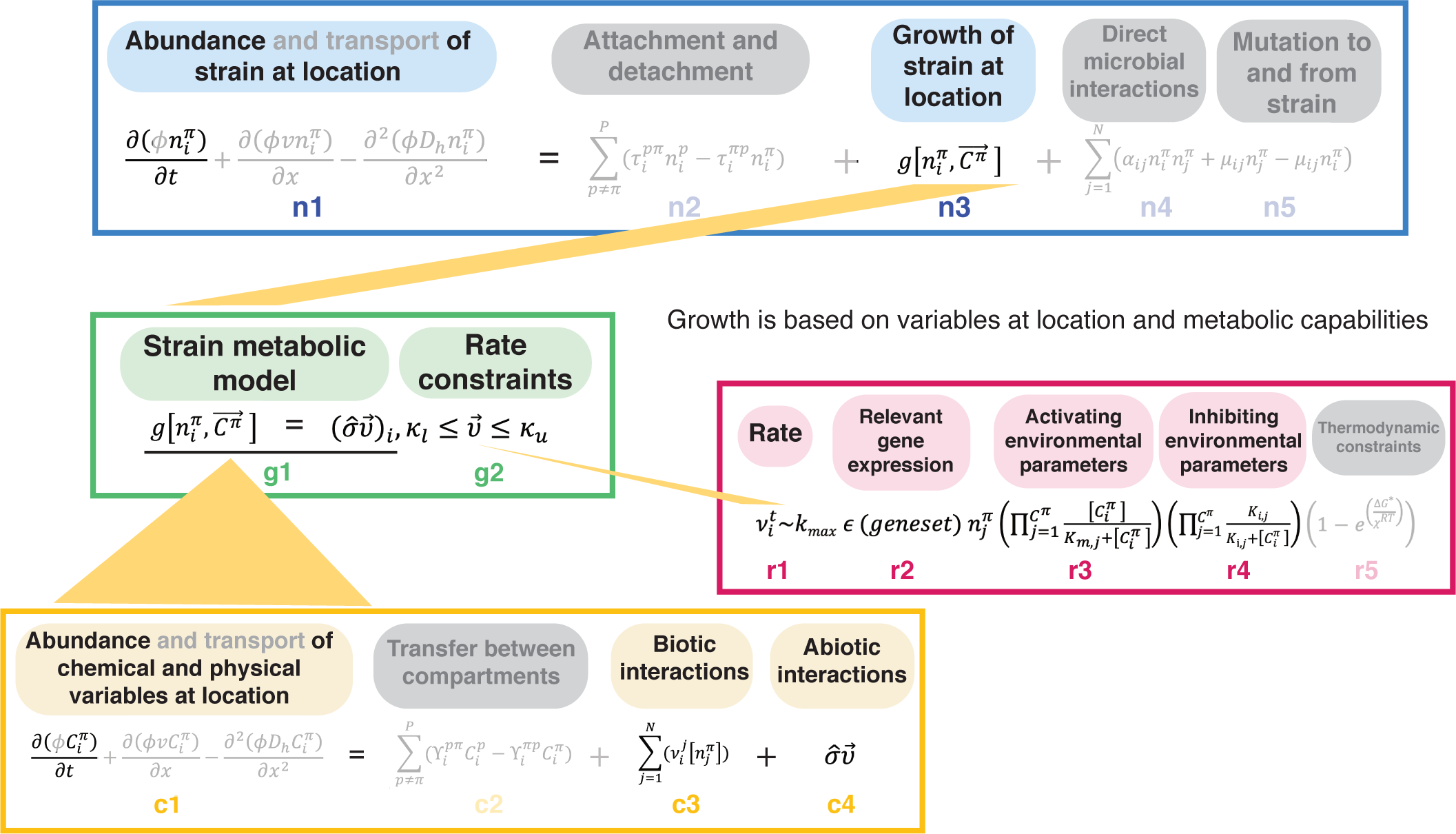


**Supplementary Figure 1**: Reproduction of FICSME framework equations with selected terms for Nitrous oxide off-gassing example study highlighted in color and unselected terms in gray.

Populate selected terms with existing data from literature, databases and previous work:

With a hypothesis formulated and terms selected, we then populate selected terms with existing data, where available and pertinent. Each individual parameter might be represented by a matrix, series of equations, or combination of several data types. The parameters, existing knowledge, limitations and data types are outlined in the tables below.

**Supplementary Table 2**.  Parameters from the selected FICSME terms n1, c1

| **Parameter** | **Existing Knowledge*** | **Limitations or Knowledge gaps?** | **Data Type** |
| --- | --- | --- | --- |
| n- abundance of each microbial species | Relative abundance & composition are site specific, predictable from geochemistry, N & S cycling microbes present | Changes over time, response to specific perturbations, accurate sediment abundance and microbial activity | Depth profiles of concentrations/abundance of each species in each location at a specific sampling event  Data result from DNA sequencing, HPLC, IC XRD, water quality probes |
| N- microbial community membership |  |  |  |
| c- concentration of each chemical species | Types & concentrations of metals, ions, pH, DO, temperature etc. Thresholds of nutrients that inhibit processes. | Changes over time, response to specific perturbations, gasses |  |
| C- all types of geochemistries present |  |  |  |
| π- which compartment n or c found in | Sediment, depth resolved | Higher resolution of sediment structure & groundwater flow |  |

**Supplementary Table 3**.  Parameters from the selected FICSME terms n3, c3, c4, g1, g2, r1-r4

| **Parameter** | **Existing Knowledge*** | **Limitations or Knowledge gaps?** | **Data Type** |
| --- | --- | --- | --- |
| ν- rates of biotic-mediated chemical transformations | Methods to measure N-cycle reactions *in situ* and *in vitro*, measured in some conditions | Not measured as compartment specific or community-level | Matrices of rate laws, their kinetic parameters, reactions rates given different environmental conditions, ability to calculate flux for one compartment.  Data results from chemistry experiments, in situ monitoring, activity assays on whole communities, cells and isolated enzymes. |
| υ- rates of abiotic chemical transformations | Have k_sp_ and other parameters for some N, S, C cycle reactions | Not known for all reactions and not in compartment specific conditions |  |
| σ- chemical reaction matrix | N&S cycle reactions, methods to calculate flux-based metabolic models based on metagenomes | Impossible to describe all, need boundary conditions & knowledge of causal factors (use of DOE) |  |
| g- specific growth rate | Measured for some ORR isolates | Not high throughput, strain, and condition variability in different environments unknown, requires isolates and good growth models properly bounded by nutrient and stressor flux constraints |  |
| k_max_, k_m_, k_i_ - enzyme kinetic constants | Measured for some N&S cycle enzymes in some ORR isolates |  |  |
| *gene set-* all of the genes | Annotated metagenomes of ORR microbial communities | Mis and un-annotated genes, proteins with multiple functions | Annotation of DNA sequencing data |
| ε- enzyme concentration and activity | N&S cycle measured in isolates *in vitro* | Community or *in vivo* measurements | qPCR, transcriptomics, quantitative proteomics |

*for literature references of existing knowledge see Box 3

Identify knowledge gaps (unpopulated or incompletely populated necessary terms):

While this is not a complete list, we list some of the knowledge gaps surrounding our target ecological process. Each knowledge gap pertains to how perturbations in the environment alter conditions and phenotype presentation in one compartment.

1. Carbon source and electron donor preferences and availability stimulating different microbes and metabolisms, specifically for nitrous oxide producing microbes.
2. Effects of low pH on NosZ enzyme which converts N_2_O to nitrogen gas on the denitrification pathway.
3. The (bio)availability of molybdenum in the compartment of interest, an essential cofactor for the nitrate reductase enzyme activity to convert nitrate to nitrite.
4. The concentration, oxidation states and form of iron, manganese, and mineral catalysts such as “green rust,” and their impact on chemodenitrification.
5. If sulfate-reducing bacteria are active in the compartment, and if the sulfide produced will shift nitrate respiration mode from denitrification to Dissimilatory Nitrate Reduction to Ammonia (DNRA) in any or all of the many types of denitrifying microorganisms.
6. If Ferric iron reducing bacteria are present in the compartment and generate ferrous iron that can influence denitrification end-products.

State specific testable sub-hypothesis for each knowledge gap:

 Considering the knowledge gaps and existing information, this combination yields specific sub-hypotheses about the concurrent contributions of abiotic and biotic factors to the target environmental phenotype. These are some hypotheses that can be tested in the first experimental cycle. We expect model results to need refinement through the iterative testing of additional hypotheses in subsequent experimental cycles.

1. Nitrous oxide production will be stimulated by influx of carbon sources and electron donors from a rainfall event.
2. Low pH inhibits the NosZ enzyme, leading to an increase in nitrous oxide production over nitrogen gas.
3. Low amounts of molybdenum will inhibit nitrate reductase enzyme activity, leading to a decrease in nitrous oxide production.
4. The concentration and oxidation states of iron and manganese will control rates of chemodenitrification and thereby the flux of nitrous oxide.
5. Biogenic sulfide will shift nitrate respiration mode from denitrification to DNRA reducing the amount of nitrous oxide produced.

Test prediction: Design and carry out experiments for each sub-hypothesis

The FICSME can be used iteratively to incorporate all hypotheses and concomitant processes and factors. For this example, one hypothesis would be evaluated at a time by changing the factors or perturbations tested at each stage of the proposed experimental cycle and then iterating as necessary. We describe one cycle that would initiate the process of determining the controls on nitrous oxide off-gassing in the ORR subsurface and how the interactions of microbes and geochemistry regulate this process. Multiple cycles would be needed to refine the model and to incorporate the diverse biotic and abiotic processes that contribute to phenotype presentation in complex environments. Researchers should determine acceptable levels of model accuracy and resolution before initiating the experimental cycle.

- Experiment 1: Survey microbial community, geochemistry and N2O produced before and after a rainfall event.
  - Rationale: Rainfall events, which are frequent and significant at the ORR, act as perturbations to the individual compartments in the subsurface bringing a change in geochemical parameters like DO, nutrients, carbon sources and metals, as well as possibly introducing new cells to the microbial community. The first step is to assess for one compartment (with replicates) the composition of the microbial community and chemical constituents and the amount of nitrous oxide effluxed *in situ*.
  - Steps: 2a1 and 2a2
  - Target terms: n1 and c1 at the field level
  - Approach: This experiment would entail more of a survey style sampling of the subsurface and groundwater to monitor: the changes in composition of the microbial community, activity of the community, other meta-scale omics measurements, concentrations of geochemical parameters, and amount of N_2_O off-gassing before and after a rainfall event. This can be a time series or two-point assay depending on budget and time. True replication *in situ* is impossible, but we would sample at least three geographically close and biogeochemically similar areas at the same depth for each replicate. Since we are starting with one compartment for this first experimental cycle, the sampling replicates would be from just one area at the ORR site.
  - Expected Outcomes: The experiment would yield amplicon and metagenomics sequencing data, concentrations and identities of chemical components, values of environmental parameters like temperature and DO. By comparing the samples collected before and after a rainfall event, the chemical and microbial species that changed in membership or abundance would be of most interest because their change in presentation is most likely related to the rainfall perturbation event. Species that respond to the perturbation are more likely to be key players in the mechanism driving phenotype presentation. With the responsive microbes and geochemistries identified, they are selected for enrichment and factor testing in Experiment 2.
- Experiment 2: Time series of enrichment in bioreactors before and after simulated rainfall, monitoring microbial populations and metabolites
  - Rationale: While a field sampling survey provides condition specific information, it does not permit replication nor investigation of effects of multiple perturbations rapidly. Therefore, the next step is to grow enrichments of the sampled microbial communities in replicate bioreactors and to quantitatively assay responses to applied system perturbations.
  - Steps: 2b1 and 2b2
  - Target terms n3, c3 and c4 at the mesocosm level
  - Approach: This experiment involves growing the enriched field communities in replicate bioreactors that attempt to mimic the geochemistry and sediment structure from the corresponding depth in the subsurface. Perturbations are quantitatively applied to the bioreactors that attempt to simulate environmental processes of interest like rainfall events. The changes to the community and chemistry are monitored with higher resolution techniques, true and more replicates, and finer time series samplings that assess the response of individual organisms, genes, proteins, and metabolites. Data types include metagenomics, transcriptomics, proteomics, metabolomics and the concentrations of chemicals from Experiment 1 like pH, temperature, nutrients and metals.
  - Expected Outcomes: The assays will pinpoint exactly which microbes and which chemicals are responsive to rainfall events and quantitate the direction and magnitude of that response. The key responsive microbes and chemicals are then isolated for even higher resolution studies in Experiment 3.
- Experiment 3: Systems analysis of field isolates responding to determined key factors
  - Rationale: Now that we know which microbes and which chemicals are responsive to rainfall event perturbations, the next step in determining causality is to decipher which genes, proteins and metabolites are active drivers of phenotype presentation and how they are acting.
  - Steps: 2c1-3
  - Target terms: g2, r1-r4 at the isolate/molecule level
  - Approach: Experiment 3 is an in-depth study of the isolated microorganisms, enzymes, metabolites or abiotic factors that have been identified in having roles leading to the off-gassing of nitrous oxide. This can include characterization of regulation, toxicity mechanisms, nitrogen metabolism, gene function and enzyme activity, all assayed in a variety of field-mimicking conditions and over time that establish the boundaries of the behavior of each molecule and microbe. The focus in this first round of the experimental cycle would be to determine which microbes (and enzymes and genes) are carrying out each step of the complete denitrification pathway and what the controls and limits are on these strains, enzymes and gene expression. Combinations of transcriptomics and metabolomics or targeted qPCR are methods to find active molecules with certain functions.
  - Expected Outcomes: The amassed knowledge from molecular reductionist studies will lead to proposing a mechanism that describes the chain of causality between the flux in biotic and abiotic factors during a rainfall event that leads to the observable phenotype in changes in the amount of N_2_O off-gassing. We will have identified the microbe responsible for carrying out each step of the complete denitrification pathway. We will then propose a mechanism for nitrous oxide production in this compartment that links microbial and chemical species and activities.
- Experiment 4: Demonstrate mechanism using a synthetic community of field isolates
  - Rationale: Since the mechanism was proposed at the isolate level and directly testing in the field is costly and inefficient, the mechanism of nitrous oxide off-gassing should first be tested quantitatively in a simulated environment of a bioreactor, like experiment 2. However, this time, the bioreactor will be populated with a synthetic community of isolates from Experiment 3 because we want to verify the mechanism by only including factors that play an active role in the target process.
  - Steps: 2b3
  - Target terms: n1, g1 and c1 at the mesocosm level
  - Approach: This experiment entails populating the same bioreactor system in Experiment 2 with a synthetic community of the isolates from Experiment 3 that collectively will simulate the environment and phenotype by carrying out complete denitrification. A perturbation is induced quantitatively to test the mechanism proposed at the conclusion of Experiment 3 and measured over time. The main measurement is the amount of N_2_O produced.
  - Expected Outcomes: If the synthetic community validates the mechanism of nitrous oxide production in the compartment at this mesocosm level, then the prediction is tested back in the field.
- Experiment 5: Induce perturbation and determine if amount and rate of nitrous oxide off-gassing prediction is correct
  - Rationale: In order to determine the mechanism at a molecular level, it was necessary to simplify the system. However, to validate the mechanism and model of rate, amount and active factors leading to N_2_O off-gassing in the ORR subsurface, *in situ* testing is required.
  - Steps: 2a2 and 2a3
  - Target terms n1, g1 and c1 at the field level
  - Approach: This experiment involves going back to the original field site in Experiment 1 and introducing a quantitative perturbation into the system and monitoring the results of the prediction based on the determined mechanism. The primary measurement would be the rate and amount of N_2_O off-gassing, as the primary target of the hypothesis and model. However, monitoring other geochemistry and the microbial community would be advisable. The perturbation would likely be an amendment of a carbon source or other nutrient meant to stimulate a certain set of microbes into carrying out complete denitrification.
  - Expected Outcomes: If the model is correct, then the known amount of amendment added should result in the predicted amount and rate of N_2_O off-gassing. If the model is not correct, the researcher should determine (before performing the experiment) what level of accuracy is sufficient for them to address their specific question. If the result is not correct, but within the boundaries, the researcher can proceed to Step 3. If the result lies outside the target boundaries, then it is necessary to re-evaluate the model and refine it by designing and performing more experiments.

Evaluate Result:

For each of the individual experiments performed, analyze the results, and see if it matched the prediction or if that knowledge gap filled. Refine as necessary by performing additional experiments or adding relevant parameters to the model. The decision process for this example has been described in each step.

**Step 3:**

Integrate data by populating terms with results from step 2:

After completing the experimental cycle, the data is integrated by populating the acquired data into the selected terms of the FICSME. The value of one or more parameters should be altered and then calculate the resulting change. This should be compared to what was observed in the experiments and the hypothesis. In the case of this example, one might consider altering the pH, carbon source and nutrient load as carried out in Experiment 2 and seeing if the amount of N_2_O measured matches the calculated amount from the model. More sophisticated computational methods for prediction testing in models should be employed to increase rigor.

**Step 4:**

Pose Mechanism:

Based on the integrated data and performance of the model during prediction testing, the researcher will pose a mechanism that explains how the contributions of the various biogeochemical factors give rise to the target phenotype. Within our system, although we have not as yet performed experiments, we could postulate that nitrate contamination is driving the low pH. The low pH is reducing the bioavailability of metals that are key denitrification enzyme cofactors and also inhibiting microbial growth, thereby decreasing N_2_O off-gassing. We could predict that if the pH is raised above 6, then the amount of N_2_O off-gassing would dramatically increase from the relief of the multi-factorial growth and respiration inhibition.

Test Mechanism:

The mechanism should be tested by performing an experiment from Step 2 where the conditions of the experiment are modulated according to the mechanism and the change, and amount and rate of change are predicted within a desired level of resolution. For this example, the synthetic community in Experiment 4 should be perturbed based on the model predictions and the amount of N_2_O produced should be compared. If, however, the prediction is not accurate or other processes need to be included, then the researcher iterates on the process.

Iterate to refine mechanism and model:

We expect that it will require more than one experimental cycle to sufficiently refine the model of nitrous oxide off-gassing for the ORR subsurface because it is a complex and hydrodynamically active environment. The mechanism and model we would generate from this first round of the experimental cycle would inform us about the process in one compartment with one community with a particular set of conditions. It might be necessary to understand the behavior in other compartments and the connections between the compartments to accurately predict the amount and rate of N_2_O off-gassing from the ORR site as a whole. This can be achieved by going back to any of the previous steps or proposing new experiments as necessary within the confines of the FICSME, including adding experiments and data types that address previously unselected terms. In this way parameters will accumulate from each cycle until sufficient model prediction resolution is obtained.

**Step 5:**

Achieve Outcome:

Once the mechanism accurately predicts the system well-enough, then the researcher can stop, if their desired outcome is knowledge of the system, or use the quantitative results from the FICSME workflow to intervene in the system to induce the outcome that solves the initial problem. In this example of nitrous oxide off-gassing, the outcome would be for the researcher to control *in situ* the metabolism of the microbial community to prevent or reduce the efflux of the potent greenhouse gas, nitrous oxide.
